# Epidemiology, risk factors, and strategies to prevent and manage poisonings due to pharmaceuticals in children in low income and low-middle income countries: A systematic review

**DOI:** 10.7189/jogh.13.04173

**Published:** 2023-12-29

**Authors:** Mary Elizabeth Mottla, Mary-Ellis Bowler, Ramin Asgary

**Affiliations:** 1 Division of Emergency Medicine, Boston Children’s Hospital, Boston, Massachusetts, USA; 2 Department of Global Health, George Washington Milken Institute School of Public Health, Washington, District of Columbia, USA; 3 Icahn School of Medicine at Mount Sinai, New York, New York, USA

## Abstract

**Background:**

There are significant disparities in the burden of disease due to poisoning between children in low- and high-income countries (HICs). However, there is limited data on the impact of increasing pharmaceutical access in low income countries (LICs) and low-middle income countries (LMICs) on the epidemiology of and risk factors associated with poisoning in children in these settings. Furthermore, while strategies in HICs have effectively reduced the burden of disease due to poisonings in children, there is limited information regarding the efficacy of these interventions in LICs/LMICs.

**Methods:**

We conducted a systematic review in eight databases for literature published between January 2000 to April 2022 to evaluate the epidemiology and risk factors associated with poisonings due to pharmaceuticals and effective strategies to prevent and manage them in children in LICs/LMICs. From 16 061 retrieved articles, 41 were included in the final analysis.

**Results:**

Pharmaceuticals were a common cause of poisoning in children in LICs/LMICs, occurring in between 12.4% and 72.36% of cases. Major risk factors were unsafe medication storage and inadequate caregiver knowledge. Delayed access to care and younger age were associated with increased mortality. Prevention strategies that included education demonstrated improvements in knowledge; however, their impact on incidence and mortality was unclear. Management strategies detailed individual patient care interventions, most commonly gastric lavage and activated charcoal. Meanwhile, delayed presentation, limited provider knowledge, and inadequate laboratory resources to support therapeutic monitoring hindered optimal management.

**Conclusions:**

The combination of educational interventions for prevention, along with regulatory processes to maximise medication storage and formulation safety, could be effective in reducing the burden of poisoning in LICs/LMICs. The development of national or regional protocols for the management of common medication poisonings, augmented by the development of poison control centers and expansion of laboratory access in facilities may help reduce the morbidity and mortality associated with pharmaceutical poisonings in children in LICs/LMICs. Further evidence regarding contextual factors, risk and benefit profiles, the pattern of poisoning, and the impact of preventive and treatment interventions specific to LICs/LMICs is needed to better refine recommendations in these settings.

**Registration:**

PROSPERO: CRD42022315686

There were 2.2 million episodes of poisoning globally in 2019, 940 000 of which occurred in those under 20 years of age [1]. Children are especially vulnerable to unintentional poisoning due to their evolving dexterity and mobility, mouthing and imitation behaviors, and limited hazard awareness [2-4]. After ingestion, a child’s inability to disclose the event can result in delayed treatment and their small body mass can lead to increased toxicity, thus contributing to increased morbidity and mortality [4].

There are significant disparities in the burden of disease due to poisoning between children in low income countries (LICs) and high-income countries (HICs), with a 5.3% case fatality rate in children due to poisoning observed in the former compared to 0.05% in the latter group – a 105-fold difference, as reported by the Global Burden of Disease 2019 study [4-6]. The most recent World Report on Child Injury Prevention described pharmaceuticals as the most common source of poisoning for children in HICs, compared to fuel and lighting chemicals in LICs [4]. Ongoing efforts have been made to increase the availability of medications worldwide [7-9]. However, the ability to analyze the impact of increased pharmaceutical access on the epidemiology of and risk factors associated with poisoning in LICs and low-middle-income countries (LMICs) is limited by the scarcity of poison control centers in those contexts and the absence of specific poisoning agents in global health databases [1,6,10,11]. Furthermore, while strategies in HICs have effectively reduced the burden of disease due to poisonings in children, there is limited information regarding the efficacy of these interventions in LICs/LMICs [5,12-23].

In this systematic review, we aimed to identify and describe the epidemiology and associated risk factors of and effective strategies for preventing and managing poisonings due to pharmaceuticals in children in LICs/LMICs.

## METHODS

We registered this protocol in the International Prospective Register of Systematic Reviews (PROSPERO: CRD42022315686) and followed the Preferred Reporting Items for Systematic Reviews and Meta-Analyses (PRISMA) guidelines in designing and performing the study [24].

We systematically searched PubMed, Embase, Scopus, Web of Science, CINAHL, PsycInfo, Global Index Medicus, and the Cochrane Library for literature published between January 2000 and April 2022. These databases were last accessed in May 2022. We likewise performed supplemental searches in Google Scholar and gray literature, and manual searched the reference lists of included articles. Through discussions with experts and the assistance of an academic librarian, we used keywords and medical subject heading terms such as “poisoning”, “child”, “pharmaceutical” or “intervention”, and (LICs/LMICs name), among others (Appendix S1 in the **Online Supplementary Document**).

We included any article that presented primary data regarding the epidemiology, risk characteristics, prevention strategies, and/or management interventions concerning pharmaceutical poisonings among children and/or adolescents in a LICs/LMICs, and that presented measurement of any related outcome. Pharmaceuticals were defined as medications prescribed or used for the treatment of a condition. Children and adolescents included those aged 0-19, as per the World Health Organization (WHO) definition [25]. Categorisation as an LICs/LMICs was determined by World Bank income classification at the time of data collection [26]. We excluded articles if they did not describe poisoning due to pharmaceuticals, were non-human or theoretical studies, had less than two subjects, were expert opinions or perspective pieces, or were in a language other than English or Spanish. Two reviewers independently reviewed titles and abstracts of all articles from the initial search, then independently conducted full-text reviews of remaining articles. Disputes between reviewers were resolved by a third reviewer.

Due to wide variation in study designs, we used the Joanna Briggs Institute (JBI) critical appraisal toolkit was used for risk of bias and quality assessment [27], with two reviewers independently evaluating articles using a standardised JBI checklists appropriate for each study design. Discrepancies in quality assessment scoring between reviewers were resolved through discussion. The adequate sample size threshold for epidemiologic cross-sectional studies was 246, calculated using Cochran’s equation with a 95% confidence interval (CI) and projected prevalence of 20%. Given the heterogeneous quality of epidemiologic and risk factor studies, publications reporting these topics were included if they attained a quality score ≥7. Due to their limited number, all intervention studies were included. The final quality assessment scores for all studies and a list of references of excluded studies are presented in Appendix S2 and S3 in the **Online Supplementary Document**, respectively.

We developed an adapted data extraction tool for this study to extract relevant data [28], including: year of publication, study setting, study design, sample size, participant age ranges, number of poisonings due to pharmaceuticals, and risk associated with incidence and or morbidity of pharmaceutical poisonings (for epidemiological and risk factor studies). We recorded the most common pharmaceuticals responsible for poisoning when reported. For intervention studies, data extraction also included intervention description, outcome measures, barriers, and study recommendations. If a study was missing information or information was unclear, reviewers discussed discrepancies before incorporation into the data extraction tool and only used available data in the analysis. We applied a recommended risk of bias and quality assessment for this review as described above. We hypothesised that the reasons for missing data for individual primary data studies included in this review varied widely.

## RESULTS

**Figure 1:**
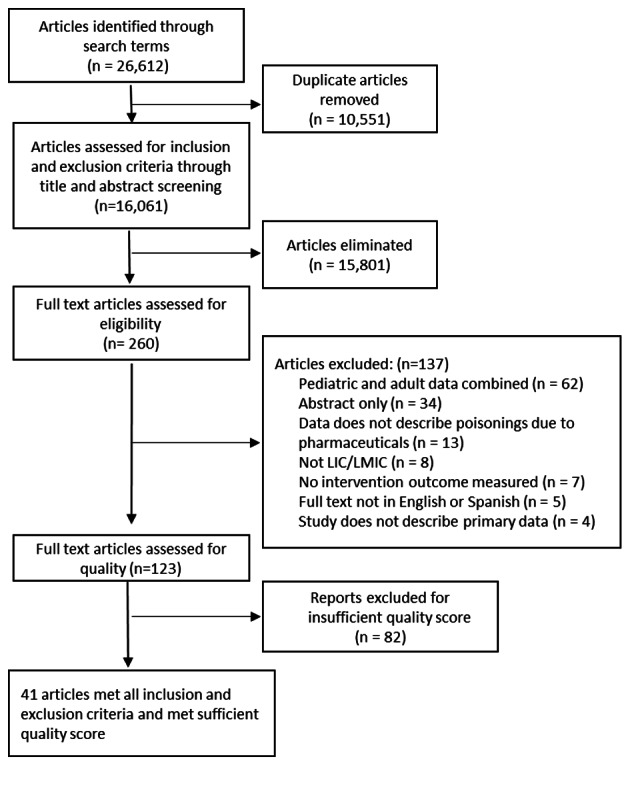
PRISMA diagram.

The database search yielded 16 061 unique articles. After deduplication, screening, full-text review, and the JBI quality assessment, 41 articles were included in this systematic review (**Figure 1**). Twenty-nine articles addressed the epidemiology and/or risk factors associated with poisoning due to pharmaceuticals and achieved a quality score ≥7, five described interventions to prevent poisoning, and seven described poisoning management. In total, 30 of the studies took place in LMICs and eleven in LICs. All WHO regions were represented; however, most studies were from the South-East Asian (n = 18) and the Eastern Mediterranean regions (n = 12). There was considerable heterogeneity between studies regarding design, study participants, content, delivery of interventions, and follow-up periods. Similar interventions were synthesised, and a qualitative analysis was performed by comparing the interventions.

The 18 studies that described the epidemiology of poisoning due to pharmaceuticals among children in LICs/LMICs were cross-sectional in design and used information from hospital records, poison center data, or forensic autopsies (**Table 1**) [29-46]. Two studies described intentional poisonings only, four studies both intentional and unintentional, and the remaining twelve did not differentiate between the two [29-46]. The percentage of poisonings due to pharmaceuticals ranged from 12.4% to 72.36% [39,42]. Commonly implicated medications in these studies included analgesics/antipyretics, antipsychotics, antiepileptics, sedatives and nutritional supplements.

**Table 1:** Epidemiology

Author (year)	Title	Country and income status	Setting	Study design	Age range in years	Number of subjects	Percentage of poisoning due to pharmaceuticals	Pharmaceuticals implicated
Intentional								
Arslan et al. (2007) [29]	Suicide among children and adolescents: Data from Cukurova, Turkey	Turkey (LMIC)	Forensic autopsies	Cross-sectional	0-18	186	32.8	Not described
Senarathna et al. (2012) [30]	Changing epidemiologic patterns of deliberate self-poisoning in a rural district of Sri Lanka	Sri Lanka (LMIC)	Multiple hospitals within a province	Cross-sectional	12-19	1100	31.36	Paracetamol
Intentional and un-intentional								
Hamid et al. (2005) [31]	Acute poisoning in children	Pakistan (LIC)	National referral center	Cross-sectional	0-15	346	Accidental: 48.6, intentional: 2.3, total: 50.9	Antidiarrheals, antihistamines, antiemetics, hypnotics/sedatives, opiates
Prasadi et al. (2018) [32]	Paediatric poisoning in rural Sri Lanka: an epidemiological study	Sri Lanka (LMIC)	Multiple hospitals within a district	Cross-sectional	0-12	422	Accidental: 15.9, intentional: 5.2, unknown: 0.2, total: 21.3	Paracetamol
Wananukul et al. (2007) [33]	Human poisoning in Thailand: The Ramathibodi Poison Center's experience (2001-2004)	Thailand (LMIC)	Poison Control Center data	Cross-sectional	0-12	2601	Accidental: 28.8, intentional: 1.2, unknown: 0.5, total: 30.5	Antipyretics (mainly paracetamol), tranquilisers
Wang et al. (2017) [34]	Epidemiologic characteristics of poisoning among hospitalised children in Shanxi, a north Chinese city during 2008-2013	China (LMIC)	Multiple tertiary care hospitals	Cross-sectional	0-14	378	Accidental: 63.2, intentional: 2.1, total: 65.3	Not described
Un-specified poisoning								
Azab et al. (2016) [35]	Epidemiology of acute poisoning in children presenting to the poisoning treatment center at Ain Shams University in Cairo, Egypt, 2009-2013	Egypt (LMIC)	Poisoning treatment center	Cross-sectional	0-18	38 470	40.21	Analgesics and antipyretics, antirheumatics, respiratory, antimicrobials, opiates
Dayasiri et al. (2018a) [36]	Patterns and outcome of acute poisoning among children in rural Sri Lanka.	Sri Lanka (LMIC)	Multiple tertiary care hospitals	Cross-sectional	9 mo-12 y	1621	25.29	Paracetamol
Diallo et al. (2021) [37]	Epidemiology of poisoning in Bamako, Mali	Mali (LIC)	Multiple reference health centers	Cross-sectional	0-14	507	43.59	Not described
Farag et al. (2021) [38]	Pattern of acute pediatric poisoning at Banha poisoning control center, Egypt: One-year prospective study	Egypt (LMIC)	Tertiary care teaching hospital	Cross-sectional	0-18	755	46.89	Not described
Gheshlaghi et al. (2013) [39]	Acute poisoning in children; a population study in Isfahan, Iran, 2008-2010	Iran (LMIC)	Tertiary care teaching hospital	Cross-sectional	0-9	322	72.36	Opiates, acetaminophen, benzodiazepines, vitamins, antibiotics
Gupta et al. (2003) [40]	A study of childhood poisoning at National Poisons Information Centre, All India Institute of Medical Sciences, New Delhi	India (LIC)	Poison Control Center data	Cross-sectional	0-18	948	23	Anticonvulsants, benzodiazepines, thyroid hormones, analgesics, iron
Lucas et al. (2006) [41]	A hospital based prospective study of acute childhood poisoning.	Sri Lanka (LIC)	National referral hospital	Cross-sectional	0-12	1888	32.9	Topical medications, anticonvulsants, psychiatric, medications
Sil et al. (2016) [42]	A study on clinico-epidemiological profile of poisoning in children in a rural tertiary care hospital	India (LMIC)	Tertiary care hospital	Cross-sectional	0-12	393	12.4	Acetaminophen, sedatives, iron, antipsychotics, antihistamines
Traore et al. (2018) [43]	Descriptive study of acute poisoning cases admitted to Yalgado Ouedraogo University Hospital in Ouagadougou, Burkina Faso	Burkina Faso (LIC)	Tertiary care hospital	Cross-sectional	0-15	253	45.9	Antimalarials, traditional medications, analgesics, antibiotics, hypnotics/sedatives
Winston et al. (2017) [44]	Drug poisoning in the community among children: a nine years' experience from a tertiary care center in south India	India (LMIC)	Tertiary care hospital	Cross-sectional	0-16	997	33.7	Antiepileptics, CNS depressants, psychotropic agents, analgesics, bronchodilators
Z'gambo et al. (2016) [45]	Pattern of acute poisoning at two urban referral hospitals in Lusaka, Zambia	Zambia (LMIC)	Multiple referral hospitals	Cross-sectional	0-19	253	21.74	Analgesics, antibiotics, antipsychotics, antimalarials, nutritional supplements
Renu et al. (2017) [46]	A portrayal of childhood injury among under-five y children	India (LMIC)	Household surveys	Cross-sectional	Caregivers of 0-5 y olds	2040	Prevalence of accidental ingestion of medicine kept in the house 3.0 (95% CI = 2.0-3.4)	Not described

CI – confidence interval, CNS – central nervous system, LIC – low income country, LMIC – low-middle income country

Twelve studies investigated risk factors associated with pharmaceutical poisonings (**Table 2**) [31,47-57]; five included both intentional and unintentional poisonings, while the remainder described unintentional studies in young children [31,49,50,52,57]. The unsafe storage of medications, variably defined as storage in an unlocked location, storage at a height accessible by children, and/or storage in unlabelled or incorrectly labeled containers, was assessed as a risk factor for poisoning in seven of these twelve studies [31,47-51,56]. Four of these seven studies conducted statistical analyses comparing caregivers of children who presented to a medical facility for poisoning with caregivers of age and gender matched controlled children who presented for reasons other than poisoning. All four studies found that caregivers of the poisoned cases were more likely to report that medication was stored in an unsafe location in the home compared to controls (odds ratio (OR) = 2.85-16.59; *P* < 0.05), with reported associated population attributable risk of 12% [47,48,51,56]. The remaining three studies that investigated storage as a risk factor interviewed caregivers of children who had been poisoned, but did not compare them with a control group to calculate a risk statistic [31,49,50]. Dayasiri et al. (2018) [50] conducted a qualitative study detailing caregiver endorsement of unsafe medication storage, while Dayasiri et al. (2020) [49] and Hamid et al. [31] presented the prevalence of self-reported unsafe storage by the caregivers of children who presented for poisoning as 43.8% and 47.8%, respectively.

**Table 2:** Risk factors

Author (year)	Title	Country and income status	Setting	Study design	Population	Number of subjects	Risk factors
Ahmed et al. (2011) [47]	Population attributable risk of unintentional childhood poisoning, in Karachi, Pakistan	Pakistan (LIC)	Multiple tertiary care hospitals	Case-control	Cases: children under five who presented to care for poisoning, controls: gender and age matched who presented for care within 48 h	120 cases, 360 controls	Increased risk of ingestion associated with unsafe storage of chemicals and medications in the home (height of <2 m and unlocked), associated PAR = 12%*
Ahmed et al. (2011) [48]	Predictors of unintentional poisoning among children under five years of age in Karachi: a matched case-control study	Pakistan (LIC)	Multiple tertiary care hospitals	Case-control	Cases: children under five who presented to care for poisoning; controls: gender and age matched children who presented for care for alternative complaints within 48 h	120 cases, 360 controls, (32.5% pharmaceutical poisonings)	Increased risk of ingestion associated with unsafe storage of chemicals and medications in the home (height of <2 m and unlocked), mOR = 5.6 (95% CI = 1.9-16.7)†
Dayasiri et al (2020) [49]	Accidental and Deliberate Self-Poisoning with Medications and Medication Errors among Children in Rural Sri Lanka	Sri Lanka (LMIC)	36 rural hospitals	Cross-sectional	Children poisoned by pharmaceuticals who presented for medical care at rural hospitals aged nine months to12 y	410	Of 16 patients poisoned by a caregiver: 43.8% due to unsafe storage, 37.5% due to inadequate parental knowledge; no significance testing done
Dayasiri et al. (2018b) [50]	A qualitative study of acute poisoning related emergencies in the pediatric age group	Sri Lanka (LMIC)	Rural tertiary care hospital	Cross-sectional survey	Caregivers of children aged nine months to 12 y hospitalised due to poisoning	383, (29.2% pharmaceutical poisonings)	Unsafe storage of medications associated with risk of ingestion; delay in care associated with ingestion of a family member’s medication; physical and economic barriers to reaching health care facilities led some to attempt forceful vomiting; no significance testing done
Dayasiri et al (2017) [51]	Risk Factors for Acute Unintentional Poisoning among Children Aged 1-5 Years in the Rural Community of Sri Lanka.	Sri Lanka (LMIC)	Rural tertiary care hospital	Case-control	Cases: children one to five years who presented to hospital with unintentional poisoning; controls: gender and age matched children who presented to for care for alternative complaints	300 cases, 300 controls, (29.3% pharmaceutical poisonings)	Increased risk of ingestion associated with unsafe storage of medications in the home, OR = 2.85 (95% CI = 2.04-4.00)
Gholami et al. (2022) [52]	Fatal outcome in acutely poisoned children with hospitalisation a 10-y retrospective study from Tehran, Iran	Iran (LMIC)	Referral hospital for poisoning	Cross-sectional	Children aged 0-12 who died from poisoning	24, (83.3% pharmaceutical poisonings)	Majority of mortalities (80%) occurred in children under five, all deaths in children <1 y were from raw opiates given by family members; no significance testing done
Haidar et al (2020) [53]	Suspected paracetamol overdose in Monrovia, Liberia: A matched case-control study	Liberia (LIC)	Paediatric referral hospital	Case-control	Cases: hospitalised children one month to five years with ≥2 symptoms of multiorgan failure consistent with paracetamol overdose, two Pooled control groups of children admitted, and those not admitted to the hospital	30 cases, 53 hospital controls, 48 community controls	Increased likelihood of inaccurate paracetamol dosing by parents in cases compared to controls (pooled: aOR = 6.6, 95% CI = 2.1-21.3; hospital: aOR = 3.7, 95% CI = 1.0-13.1)
Hamid et al. (2005) [31]	Acute poisoning in children	Pakistan (LIC)	Tertiary care teaching hospital	Cross-sectional	Children one month to 15 y in hospital	346	Unsafe storage reported in 47.8% of subset of 90 patients with additional clinical information available; increased mortality for children from peri-urban slums (*P* = 0.001); increased mortality for children <6 mo (*P* = 0.012)
Maior et al. (2020) [54]	Demographics, deaths and severity indicators in hospitalisations due to drug poisoning among children under age five in Brazil	Brazil (LMIC)	National hospital data	Cross-sectional	Children <5 y hospitalised for drug poisoning	17 725	Higher death to hospitalisation rate seen in the region with fewer health care facilities and more geographic barriers to travel, attributed to delay in arriving to care facility; no significance testing done
Ramana- yake et al. (2012) [55]	Knowledge and practices of paracetamol administration among caregivers of pediatric age group patients.	Sri Lanka (LMIC)	Outpatient clinic	Cross-sectional survey	Caregivers of children aged 0-11 y of age who presented to family practice clinic for regular care	98	Inadequate parental knowledge resulting in supratherapeutic doses was common (43%), lack of knowledge regarding differences in strength between adult, child, and infant forms of medication (50%); no significance testing done
Ramos et al. (2010) [56]	Risk factors contributing to childhood poisoning	Brazil (LMIC)	Multiple emergency departments	Case-control	Cases: caregivers of children under 60 mo of age seeking care after accidental ingestion of poisonous agent; controls: caregivers of age- matched children seeking care for alternative complaints	Cases: 25, controls: 25, (72% pharmaceutical poisonings)	Increased risk of ingestion associated with unsafe storage of toxic agents below 150cm, aOR = 16.59 (95% CI = 2.86-96.20), attributable fraction 19%
Shadnia et al. (2013) [57]	Methadone toxicity: Comparing tablet and syrup formulations during a decade in an academic poison center of Iran	Iran (LMIC)	Poison referral hospital in capital city	Cross-sectional	Children aged 0-18 who presented for care with documented methadone poisoning due to tablet or syrup formulations	292	Increased likelihood of ingestion of syrup compared to tablet for children <12 y (29.7% vs 10.4%, *P* < 0.001)

aOR – adjusted odds ratio, CI – confidence interval, h – hours, LIC – low income country, LMIC – low-middle income country, m – meters, mo – months, mOR- matched odds ratio, PAR – population attributable risk, y – years

*PAR = (probability of exposure to disease(matched odds radios-1))/matched odds ratio.

Five studies examined inadequate parental medical knowledge, described as the provision of an incorrect dose, an inappropriate medication, or a harmful traditional medicine resulting in poisoning as a risk factor [49,50,52,53,55]. Baseline parental knowledge of medication dosing was investigated by Ramanayake et al. [55] in Sri Lanka, where they administered a survey to parents attending a well-child clinic and found that 50% of respondents were unable to differentiate between the varying strengths of adult, child, and infant formulations of paracetamol. Only one of the studies that examined parental knowledge reported risk statistics regarding poisoning due to inadequate parental knowledge. Haider et al. [53] compared the reported doses of paracetamol given by parents between children hospitalised for suspected paracetamol hepatotoxicity with children hospitalised for other causes of hepatotoxicity and found that caregivers in the poisoning group were more likely to report having administered supratherapeutic doses (OR = 6.6; *P* < 0.05). Two additional studies described the percentage of poisoning cases in which the cause was attributed to caregiver medication errors but did not compare to controls. Dayasiri et al. [49] reported that 37.5% of all poisoning cases were associated with inadequate parental knowledge, and Gholami et al. [52] reported that 100% of poisoning deaths in children under the age of one due to administration of raw opium as a home remedy by a caregiver. The final study that included parental knowledge as a risk factor for poisoning was qualitative in nature by Dayasiri et al. [50], in which informant interviews detailed caregiver unawareness of the danger that medications prescribed for adults posed to their children.

Three studies investigated risk factors associated with differences in morbidity and/or mortality due to pharmaceutical poisonings. Two studies assessed age as a risk factor for mortality. Hamid et al. found that those less than six months had higher fatality rates compared to other age groups up to 15 years (*P* < 0.012) [31]. Gholami et al. reported that of the 24 fatal cases among children aged 0-12, 80% occurred in children under five, however the study did not calculate the significance of this statistic [52]. Three studies assessed the location of a child’s home as a risk factor for morbidity and/or mortality. A review of Brazilian national hospital data by Maior et al. [54] described children aged 0-5 who presented after poisoning, and demonstrated a higher death to hospitalisation ratio among children from geographically remote regions (0.4% nationally compared to 1.1% and 0.6% in select remote regions, no significance testing done). Authors concluded that delayed access to care resulted in these differences in mortality due to an interplay of the geographic remoteness leading to prolonged travel times, fractured health infrastructure systems, and low socioeconomic resources [54]. Dayasiri et al. [50]in Sri Lanka reported that delayed presentation to care was associated with increased morbidity and mortality among children in their qualitative study, but did not provide statistics regarding this association. Reasons for delayed access to care included geographic and socioeconomic barriers, as well as caregiver ignorance regarding the toxicity of an ingested agent. Hamid et al. [31] found that children from peri-urban slums in Pakistan had higher rates of mortality compared to children who came from urban or rural homes; however, they did not detail or postulate as to why this association existed.

Shadnia et al. [57] attempted to detail differences in morbidity and mortality after poisoning due to methadone syrup compared to tablets but did not find statistically significant differences between the two groups. Their final morbidity and mortality statistics, however, included both adults and children, and authors did not present the calculated differences for the pediatric group alone. Results from this study did reveal that children under 12 were significantly more likely to have ingested methadone syrup compared to tablet (29.7% vs 10.4%; *P* < 0.001).

We identified five interventions to prevent pharmaceutical poisonings in children (**Table 3**). Rehmani and LeBlanc [61] conducted a randomised controlled trial in Pakistan comparing a home visit program to prevent poisoning in children against a control home visit program that aimed to prevent falls in children. After the trial, more households in the poisoning prevention group, which received education and assistance to remove poisoning hazards, were classified as homes safe from possible unintentional poisoning compared to homes that received the fall prevention intervention (18.75% vs 7.2%; *P* < 0.001) [61]. Justin and Shobha [62] conducted educational home visits in India and found statistically significant improvements in knowledge, attitude, and practice (KAP) scores among participants after the intervention (*P* < 0.001). Foulds et al. [58] and Mandiracioglu et al. [59] conducted school-based educational programs for children and caregivers in Bangladesh and Turkey, respectively [58,59]. Both studies demonstrated statistically significant improvements in post-test poisoning prevention knowledge (*P* < 0.0001 [58]; *P* < 0.05 [59]). Mohamad et al. [60] carried out an educational injury prevention program for nursery school teachers in Egypt; their post-intervention tests demonstrated improvement in teacher knowledge (*P* = 0.0001) and skill regarding poisonings (*P* = 0.001). Although all five included studies that described poisoning prevention interventions demonstrated statistically significant improvements in home safety and/or post-test knowledge, no study attempted to measure a change in the incidence, morbidity, or mortality associated with poisonings in children after their interventions [58-62]. By using the Grades of Recommendation, Assessment, Development and Evaluation Working Group (GRADE) approach, we interpreted the level of certainty regarding an expected increase in post-intervention knowledge regarding poisoning after educational interventions in LICs/LMICs as moderate certainty. This assessment was made after incorporating concerns regarding publication bias in these settings, as well as risk of bias associated with lack of control groups in these studies.

**Table 3:** Prevention

Author (year)	Title	Country and income status	Setting and population	Study design	Number of subjects	Intervention description	Outcome measurement and outcomes	Barriers to intervention	Recommendation
Foulds et al. (2021) [58]	Play safe with Sisimpur: an evaluation of a child injury prevention intervention in Bangladesh	Bangladesh (LMIC)	Schools: 3-8 y old children and adult caregivers	Quasi experimental	1288 children, 750 adults	Multi-media school based educational program	Knowledge scores: child knowledge – what to do before drinking unknown liquid from a bottle (*P* < 0.001); adult knowledge – how to store dangerous substances (*P* < 0.001)	No specific barriers described by authors	Multimedia intervention targeting both children and adults can be valuable
Justin et al. (2014) 59]	Impact of Educational Intervention on Knowledge, Attitude and Practice among General Public Regarding Accidental Poisoning	India (LMIC)	Households: adults in communities with high incidence of accidental poisonings	Quasi experimental	109	One on one educational sessions	KAP scores: pre-intervention KAP score 20.7; immediate post-intervention KAP score 39.8 (*P* < 0.001); one month follow up KAP score 34.5 (*P* < 0.001)	Requires ability to travel to participant homes, may be difficult in rural areas or with limited staff	Given drop in knowledge from first to second follow-up there is a need for continuous, ongoing education
Mandiracioglu et al. ( 2003) [59]	The Knowledge and Attitude of Rural Children about Poisonings	Turkey (LMIC)	Rural primary schools: elementary students and parents	Quasi experimental	247	School based educational program	KAP scores: increase in child awareness of the existence of a poison information center from 27.9% to 62.9% (*P* < 0.05); students’ KAP scores increased from 21.2 to 40.6 (*P* < 0.05); parents: 98.5% of parents read the booklet within two weeks of distribution	Although there was a national poison control center, no regional center available as resource for rural communities	Increase the availability of preventive training, especially in rural areas; establish regional poison information centers
Mohamad et al. (2018) [60]	Effect of Accident Prevention Program on Nursery School Teachers knowledge and Practices	Egypt (LMIC)	Urban nursery schools: teachers	Quasi experimental	60	Lectures and interactive skills stations	KAP scores: pre-intervention knowledge 0.72; immediate post intervention knowledge 1.00, one month follow up 0.98 (*P* = 0.0001); pre-intervention first-aid skill increased from 1.15 pre-test to 5.73 immediately post-test, and 5.43 at the three month follow up (*P* = 0.001)	Fairly significant time commitment, and requires financial investment for equipment	Course content for student teachers should include training with lectures; school authority should provide teachers with informational handouts as well as equipment to improve knowledge and practices about accident prevention and first aid
Rehmani et al. (2010) [61]	Home visits reduce the number of hazards for childhood home injuries in Karachi, Pakistan: A randomised controlled trial	Pakistan (LIC)	House-hold level: parents of children <3 y	RCT	304 (151 in ingestion group)	Families were randomised to receive one on one poisoning or fall educational intervention	KAP Scores and behavior change: 18.75% of previously unsafe ingestion homes became safe after intervention compared to 7.2% in control group (*P* < 0.001); mean number of ingestion hazards decreased from 2.3 to 1.9 (*P* < 0.001)	Entered 88% of homes, depending on community may be more or less difficult to gain access	Home visiting programs should be tested across different communities to determine utility (one size does not fit all); Pakistan benefits from large cadre of female community health workers - would be useful to maximise this work force

KAP – knowledge, attitudes, and practices, LIC – low income country, LMIC – low-middle income country, y – years

Seven studies described interventions to manage poisonings (**Table 4**). Two quasi-experimental educational interventions in India and Egypt aimed to improve first aid knowledge among adolescents; both demonstrated statistically significant improvements in post-intervention knowledge scores [63,64]. Neither study attempted to measure changes in poisoning morbidity or mortality after the respective interventions.

**Table 4:** Management

Author(year)	Title	Country and income status	Setting and population	Study design	Number of subjects	Intervention description	Outcome measurements and reported outcomes	Barriers	Recommendations
Das et al. (2020) [63]	A study on the effectiveness of educational intervention regarding first aid management of selected medical emergencies among adolescents at a school in Kolkata	India (LMIC)	School: children 14-19	Quasi experimental	201	School-based lecture and practical sessions	KAP scores: pre-intervention knowledge regarding poisoning management: 17.9%; post-intervention: 65.7% (*P* < 0.001)	School-based study- taking class time may be difficult to replicate	Repeated, hands-on on and interaction educational sessions
Wafik et al. (2014) [64]	Effectiveness of a first-aid intervention program applied by undergraduate nursing students to preparatory school children	Egypt (LMIC)	School: children 11-16	Quasi experimental	100	School-based lecture and practical sessions led by nursing students	KAP Scores: students with satisfactory knowledge of first aid regarding poisoning: 5% pretest, 98% post-test, and 99% at follow up test two months later (*P* < 0.001); students with adequate first aid skill pretest = 0%, post-test: 91%, follow up 59% (*P* < 0.001)	Decline in knowledge and practice scores over time	Repeated practice sessions should be held so as not to lose skills
Bek et al. (2008) [65]	Charcoal hemoperfusion in amitriptyline poisoning: experience in 20 children	Turkey (LMIC)	Tertiary care hospital: children 1-15 with amitriptyline poisoning	Case series	20	Charcoal hemoperfusion	Clinical progression: all patients returned to consciousness during or immediately following hemoperfusion; all patients had resolution of abnormal electrocardiogram findings; all able to discontinue mechanical ventilation	Early management crucial; 95% of patients received within 1-2 h of presentation	If cardiac or respiratory compromise seen in patient with amitriptyline overdose, consider early charcoal hemoperfusion
Eddleston et al. (2007) [66]	The hazards of gastric lavage for intentional self-poisoning in a resource poor location.	Sri Lanka (LMIC)	Multiple hospitals: children 15-18 who received gastric lavage after intentional ingestion	Case series	3	Gastric lavage	Procedure details and clinical progression: significant physical restraint; large volume of fluid administration for all patients with significant gagging and coughing; airway not preserved in any of the patients; all patients aspirated and were started on IV antibiotics after procedure	N/A	Sri Lankan national guidelines call for intubation prior to lavage if at risk for aspiration, minimal physical restraint, and low volume lavage. These were not followed in these cases, recommend additional education for providers.
Musumba et al. (2004) [67]	Salicylate poisoning in children: Report of three cases	Kenya (LIC)	District hospital: children 3-4 who presented with salicylate poisoning	Case series	2	Sodium bicarbonate	Clinical progression: case #1 died due to cardiopulmonary arrest (presented 48 h after ingestion); case #2 regained consciousness after infusion and was discharged after three days (presented 24 h after ingestion)	Often unable to measure salicylate levels which limited the ability to manage resuscitation	Increased access to timely laboratory access; accurate labeling of salicylate containing products in shops
Ozdemir et al. (2011) [68]	Fatal poisoning in children: acute colchicine intoxication and new treatment approaches	Turkey (LMIC)	Tertiary care hospital: children 0-16 with colchicine poisoning	Case series	23	Medication administration depending on dose ingested	Clinical Progression: children who ingested lower doses of colchicine all survived (received activated charcoal/gastric lavage); one low dose poisoning case required intensive management, associated with delayed presentation; lethal dose poisoning case began therapy within 45 min of ingestion and survived without lasting effects. Two other children with poisoning ≥1.0mg/kg both died	Early administration of interventions after ingestion is imperative, delay can lead to increased morbidity and mortality	Any patient suspected of having ingested colchicine should be started immediately on fluid/electrolyte resuscitation, and consider activated charcoal/gastric lavage
Singhi et al. (2003) [69]	Acute iron poisoning: clinical picture, intensive care needs and outcome	India (LIC)	Tertiary care hospital	Case Series	21	Medication administration	Clinical progression: all received gastric lavage; IV deferoxamine given to 16; often unable to obtain serum iron levels, decision to pursue chelation therapy supported by proxy of presenting symptoms, limited close management of resuscitation; majority of patients arrived <6 h of ingestion, one who presented on day four died	Team unable to procure deferoxamine for one patient; limitations in timely laboratory results regarding iron levels and management monitoring	All children ingested bright colored sugar-coated iron tablets given to their mothers by the Reproductive and Child Health program; increased education through this program should be initiated to prevent these poisonings

h – hours, kg – kilograms, LIC – low income country, LMIC – low-middle income country, mg – miligrams, min – minutes

The five included medical management studies were case series describing interventions for iron, salicylate, colchicine, amitriptyline, and general poisonings. A case series in Kenya by Musumba et al. [67] applied sodium bicarbonate for salicylate poisoning in two preschool aged children; the authors reported they were limited in their management due to the inability to attain accurate and timely laboratory results to measure salicylate levels and monitor acid-base status during sodium bicarbonate administration. After review of the poisonings in this study, they recommended improved medication labeling as the ingested products were not labeled as containing salicylates [67]. Bek et al. [65] reported a case series of 20 patients in Turkey which detailed the use of charcoal hemoperfusion in addition to activated charcoal and gastric lavage to treat severe amitriptyline poisoning. In this study, all patients who received charcoal hemoperfusion had resolution of abnormal electrocardiogram findings and regained consciousness; all patients were treated within 1-2 hours of ingestion, and investigators thus recommended early recognition of severe poisoning and initiation of management [65].

Ozedmir et al. [68] described a case series of 23 children in Turkey with colchicine poisoning who received a multitude of medical interventions, most frequently intravenous fluids, activated charcoal, and gastric lavage. The 16 children with low dose ingestion all recovered completely after administration of gastric lavage and/or activated charcoal; however, one child required more intensive interventions after presenting 20 hours after initial exposure. Alternatively, one child who ingested a presumed lethal dose of colchicine survived after presenting within 45 minutes. The authors recommended early recognition and management for patients suspected of having ingested colchicine [68].

Singhi and Baranwal [69] described the medical management of 21 children with iron poisoning in India. All children received gastric lavage and 16 received chelation therapy with deferoxamine. The decision to initiate and subsequent management of chelation therapy was difficult due to laboratory limitations that restricted the ability to obtain timely and accurate iron serum levels [69]. While most patients presented within six hours and survived, one was brought to care four days after poisoning and later passed away. All children had ingested bright colored sugar-coated prenatal tablets, leading the authors to recommend adjustment in the formulation of these tablets and/or improved education for tablet recipients [69].

A small case series of three adolescents in Sri Lanka by Eddleston et al. [66] described the use of gastric lavage for intentional poisonings. They found a lack of appropriate monitoring or patient safety measures, as well as improper variations in the quantity and quality of fluid used in the lavage, with subsequent poor outcomes including emergent intubation and aspiration described. Although national guidelines for the use of gastric lavage exist in Sri Lanka, many of the cases deviated from these protocols, so the authors recommended improved education for providers [66].

## DISCUSSION

The studies included in this review described variable data and evidence regarding epidemiology, risk factors, and prevention or management strategies of pharmaceutical poisoning among children in LICs/LMICs. Of the eighteen epidemiologic studies, twelve reported that more than 30% of poisonings in children were due to pharmaceuticals [29-31,33-35,37-39,41,43,44]. The most common pharmaceuticals responsible for poisonings varied between studies and settings, but often included analgesics/antipyretics, antipsychotics, antiepileptics, sedatives and nutritional supplements. Improvements in poisoning surveillance in LICs/LMICs could help governments leverage limited health systems resources and prioritise management strategies for the most frequently encountered pharmaceutical poisonings in their regions [70].

Most studies concluded that there are significant differences in risk of pharmaceutical poisoning for children in households in which medications are stored safely compared to those in which they are not [47,48,51,56]. Other studies did not present comparative risks, but concluded that unsafe storage is indeed a risk factor for poisoning in children [31,49,50]. Furthermore, medical management studies recommended improved labeling and storage of medications as ways to reduce the incidence and severity of poisonings [65,67-69]. Barriers to these changes in LICs/LMICs include the costs of safe storage devices and a lack of legislation requiring child safety medication packaging [50,71]. In South Africa, researchers distributed 20 000 child-resistant paraffin containers to address limited access to safe storage equipment, and demonstrated a decrease in the incidence of accidental paraffin poisoning by 47% [72]. Pharmaceutical poisonings in children in LICs/LMICs may be reduced by employing a similar strategy and distributing child-safe containers for families [72]. Besides ensuring safe storage at home, safer medication packaging upon distribution may further reduce pharmaceutical poisonings. Shadnia et al. [57] studied differences in morbidity and mortality associated with medication formulation and demonstrated that younger children were significantly more likely to ingest the syrup formulation. Given that younger children have higher rates of mortality after poisoning, the increased risk of ingesting a syrup may be associated with a higher overall mortality risk, contributing to an elevated burden of disease due to poisoning [31,52]. Limiting the potency of over-the-counter medications, the use of blister packaging and the adjustment of a drug’s formulation to a less appealing color or taste have been effective in reducing the incidence and impact of pharmaceutical poisoning in HICs [4,71]. In LICs/LMICs, there are often gaps in policies to develop, pass, and reinforce mandatory safe medication mechanisms [31,43]. Pharmaceutical companies in LICs/LMICs could consider championing industry led safety initiatives, such as the commitment in India to blister packages [73].

Here we identified inadequate parental knowledge as a risk factor for pharmaceutical poisonings in children [49,50,52,53,55]. Prevention studies demonstrated statistically significant improvements in the knowledge of participants, yet none attempted to measure the impact on incidence or harm associated with poisoning [58-62]. Similar educational interventions conducted in HICs that have measured post-intervention poisoning rates have produced inconsistent and inconclusive results regarding the impact of these interventions [20]. Furthermore, the first aid intervention studies demonstrated knowledge improvement in their target populations but did not describe the impact on morbidity or mortality rates [63,64]. A Cochrane review of studies in 21 HICs and upper middle-income countries (UMICs) and three LMICs assessed poisoning first-aid programmes by non-medical professionals and concluded that most of the evidence regarding the effectiveness on morbidity or mortality rates was of low or very-low certainty [74]. The combination of educational programmes with interventions such as medication formulation changes and packaging safeguards may be more effective in reducing pharmaceutical poisoning events and morbidity in these settings [20]. National studies in LICs/LMICs are likely to be helpful in providing the data necessary to support the effect of these combination interventions on incidence and mortality rates.

Delayed access to care is a risk factor for increased morbidity and mortality by impacting the severity of poisoning and related medical management [50,54,65,68,69]. Delayed presentation was associated with inadequate caregiver knowledge as families reported that they did not immediately seek care for their children because they did not appreciate the toxic nature of the ingested agent, while some caregivers who faced barriers in accessing timely medical care attempted harmful first aid interventions at home [50]. In most HICs/UMICs, the national poison control centers are free resources through which the public can access professional recommendations after a poisoning, including recommendations on when to seek medical care and home interventions to attempt or avoid. Additionally, these centers act as data collection sources for public health surveillance and can provide management guidance to practitioners as well [75,76]. As of 2021, no LIC and only 42% of LMICs had a poison control center [10]. In 2020, consultants at the Thailand national poison control center supported health care professionals and non-medical individuals on over 29 000 poisoning cases and assisted in pharmaceutical antidote delivery [77]. In many LICs/LMICs, most of the population has access to mobile phones, which would facilitate access to poison control teleconsultation if such a resource existed [78]. While the initial establishment of these centers will certainly require substantial funding, the long-term benefits could make this approach a viable investment in low resource settings.

Medical interventional studies in this review included gastric lavage and activated charcoal [65,66,68,69]. The American Academy of Clinical Toxicology, European Association of Poison Centers, and Clinical Toxicologists recommend against the routine use of gastric lavage in HICs, and report limited data to support the use of single-dose activated charcoal after one hour of ingestion [79,80]. In limited resource areas where the only intervention option is gastric lavage or activated charcoal, providers may understandably attempt these interventions. Nationally developed guidelines may be helpful in ensuring the appropriateness and safety of these interventions considering evidence from a local context, and a different risk benefit analysis. In settings with limited provider experience with poisonings, the development of regionally appropriate and agreed upon safety protocols may help reduce the likelihood of incurring patient harm and improve patient outcomes. In Kenya national guidelines for the management of common medical conditions in children were developed with subsequent uptake of 60% by physicians in the country [81]. Guidelines require ongoing reinforcement to be appropriately followed however, which may be accomplished through the expansion of poison control centers.

There are limitations in care due to the inability to accurately diagnose poisonings and monitor the progression of therapies [67,69]. Epidemiology articles characterised several medications such as acetaminophen, iron, and salicylates, for which the identification and management of overdoses are optimised by reliable laboratory testing [36,39-42,44]. There are significant barriers to the development of quality laboratory systems in many LICs/LMICs including limited workforce, fragmented health care systems and infrastructure, insufficient training programs, and the need for robust quality control systems [82]. Addressing many of these challenges likely requires large scale investments; however, point-of-care testing for pH levels and electrolytes in low-resource settings might be useful in improving poisoning management. Such testing programmes have successfully been evaluated in Burundi to monitor liver function tests and in several South American countries to monitor coagulation after snake bites [83,84].

This review has some limitations. During the screening process, the critical appraisal tool eliminated two-thirds of the included articles, which may indicate an overall deficit of quality studies on this topic. Most excluded studies concerned epidemiology and risk factors, and were excluded due to inadequate sample size and description of study subjects. The absence of national poison control centers combined with the scarce amount of primary research published from LICs limits the geographical representation, despite all L/LMICs being eligible for inclusion in this study. Additionally, the use of the World Bank income classification as a proxy for eliciting limited resource settings may pose some limitations in generalisability of findings. Nevertheless, we attempted to comprehensively outline th state of poisoning due to pharmaceuticals from the epidemiology to medical management and policy level challenges and opportunities in L/LMICs.

## CONCLUSIONS

Current data suggests that a significant number of poisonings among children in LICs/LMICs are due to pharmaceuticals. Risk factors for increased risk of poisoning and/or increased morbidity and mortality include unsafe medication storage, packaging, limited parental or provider knowledge, and younger age as well as delayed presentation to care. Prevention and first aid educational interventions are effective in increasing knowledge and changing practices. The ultimate impact of these programs on the burden of poisoning in these settings is, however, untested. Proposed medical management of pharmaceutical poisonings in children in LICs/LMICs can be successful in reducing morbidity and saving lives, however these processes are challenged by inadequate laboratory resources and a wide variation in implementation and providers’ uptake that do not always conform to evidence-based practice guidelines. The combination of educational interventions for prevention along with regulatory processes to maximise medication storage and formulation safety could be effective in reducing the burden of poisoning in LICs/LMICs. The development of national or regional protocols for the management of common medication poisonings, augmented by the development of poison control centers and expansion of laboratory access in facilities may help reduce the morbidity and mortality associated with pharmaceutical poisonings in children in LICs/LMICs. Further evidence regarding contextual factors, local or regional risk, benefit profiles, the pattern of poisoning, and the impact of preventive and treatment interventions specific to LICs/LMICs is needed to better refine recommendations in these settings.

## Additional material


Online Supplementary Document
