# Supplementary material for: Epidemiology, risk factors, and strategies to prevent and manage poisonings due to pharmaceuticals in children in low income and low-middle income countries: A systematic review
Source: J Glob Health. 2023 Dec 29;13:04173. doi: 10.7189/jogh.13.04173 (PMC10754493; doi:10.7189/jogh.13.04173)
Supplement: Online Supplementary Document [file jogh-13-04173-s001.pdf]

Appendix 1: Complete search terms and databases

PubMed

(Poisoning [mesh] or poison* [tiab] or ingest* [tiab] or toxic* [tiab] or intoxicat* [tiab])

AND

(Infant, newborn [mesh] or newborn* [tiab] or Infant* [tiab] or infant [mesh] or child, preschool [mesh] or preschool [tiab] or child [mesh] or child* [tiab] or adolescent [mesh] or adolescen* [tiab] or pediatric* [tiab] or pediatrics [mesh] or paediatric* [tiab] or minor* [tiab] or toddler* [tiab] or kid [tiab] or youth* [tiab] or boy [tiab] or girl [tiab] or schoolchild* [tiab] or preschool* [tiab])

AND

(Prescription drugs [mesh] or nonprescription drugs [mesh] or controlled substances [mesh] or pharmaceutical preparations [mesh] or medication* or medicine* or pharmaceutical* or drug* or prescription* or Interven* or prevent* or accident prevention [mesh] or primary prevention [mesh] or secondary prevention [mesh] or tertiary prevention [mesh] or reduc* or risk reduction behavior [mesh] or harm reduction [mesh] or educat* or education [mesh] or counsel* or counseling [mesh] or supervis* or parenting or parenting [mesh] or train* or teach* or teaching [mesh] or safety or safety [mesh] or packag* or drug packaging [mesh] or label* or drug labeling [mesh] or storage or drug storage [mesh] or childproof or “child resistant” or child restraint systems [mesh] or “poison control” or treatment or management**)**

AND

(Afghanistan or Afghanistan [mesh] or Algeria or Algeria [mesh] or Angola or Angola [mesh] or Armenia or Armenia [mesh] or Azerbaijan or Azerbaijan [mesh] or Bangladesh or Bangladesh [mesh] or Belarus or Belarus [mesh] or Belize or Belize [mesh] or Benin or Benin [mesh] or Bhutan or Bhutan [mesh] or Bolivia or Bolivia [mesh] or Bosnia or Herzegovina or Bosnia and Herzegovina [mesh] Brazil or Brazil [mesh] or Bulgaria or Bulgaria [mesh] or Burkina Faso or Burkina Faso [mesh] or Burundi or Burundi [mesh] or Cabo Verde or Cabo Verde [mesh] or Cambodia or Cambodia [mesh] or Cameroon or Cameroon [mesh] or Central African Republic or Central African Republic [mesh] or Chad or Chad [mesh] or China or China [mesh] or Colombia or Colombia [mesh] or Comoros or Comoros [mesh] or Congo or Congo [mesh] or Cote Ivoire or Cote D'ivoire [mesh] or Djibouti or Djibouti [mesh] or Egypt or Egypt [mesh] or El Salvador or El Salvador [mesh] or Guinea or Guinea [mesh] or Eritrea Or Eritrea [mesh] or ESwatini or Eswatini [mesh] or Ethiopia or Ethiopia [mesh] or Fiji or Fiji [mesh] or Gambia or Gambia [mesh] or Georgia or Georgia (Republic) [mesh] or Ghana or Ghana [mesh] or Guatemala or Guatemala [mesh] or Guyana or Guyana [mesh] or Haiti or Haiti [mesh] or Honduras or Honduras [mesh] or India or India [mesh] or Indonesia or Indonesia [mesh] or Iran or Iran [mesh] or Iraq or Iraq [mesh] or Jamaica or Jamaica [mesh] or Jordan or Jordan [mesh] or Kazakhstan or Kazakhstan [mesh] or Kenya or Kenya [mesh] or Kiribati or Kiribati [mesh] or Kosovo or Kosovo [mesh] or Kyrgyzstan or Kyrgyzstan [mesh] or Laos or Lao or Laos [mesh] or Lesotho or Lesotho [mesh] or Liberia or Liberia [mesh] or Madagascar or Madagascar [mesh] or Malawi or Malawi [mesh] or Mali or Mali [mesh] or Marshall Islands or Mauritania or Mauritania [mesh] or Micronesia or Micronesia [mesh] or Moldova or Moldova [mesh] or Mongolia or Mongolia [mesh] or Morocco or Morocco [mesh] or Mozambique or Mozambique [mesh] or Myanmar or Myanmar [mesh] or Namibia or Namibia [mesh] or Nepal or Nepal [mesh] or Nicaragua or Nicaragua [mesh] or Niger or Niger [mesh] or Nigeria or Nigeria [mesh] or North Macedonia or Republic of North Macedonia [mesh] or Pakistan or Pakistan [mesh] or Philippines or Philippines [mesh] or Romania or Romania [mesh] or Russia or Russia [mesh] or Rwanda or Rwanda [mesh] or Samoa or Samoa [mesh] or Sao Tome or Sao Tome and Principe [mesh] Senegal or Senegal [mesh] or Sierra Leone OR Sierra Leone [mesh] or Solomon Islands or Melanesia [mesh] pr Somalia or Somalia [mesh] or Sudan or Sudan [mesh] or Sri Lanka or Sri Lanka [mesh] or Syria or Syria [mesh] or Tajikistan or Tajikistan [mesh] or Tanzania or Tanzania [mesh] or Thailand or Thailand [mesh] or Timor-Leste or Togo or Togo [mesh] or Tunisia or Tunisia [mesh] or Turkey or Turkey [mesh] or Turkmenistan or Turkmenistan [mesh] or Uganda or Uganda [mesh] or Ukraine or Ukraine [mesh] or Uzbekistan or Uzbekistan [mesh] or Vanuatu or Vanuatu [mesh] or Vietnam or Vietnam [mesh] or West Bank or Gaza or Middle East [mesh] or Yemen or Yemen [mesh] or Zambia or Zambia [mesh] or Zimbabwe or Zimbabwe [mesh] or Developing countries [mesh] or Developing countr* or Low income countr* or middle income countr* or “low-middle income countr*” or LMIC or “third world” or “resource poor” or “low resource” or “under resourced” or underdevelop*)

Scopus
Search W/In ArticleTitle, Abstract, Keywords: poison* OR ingest* OR toxic* OR intoxicat*
AND
Search W/In ArticleTitle, Abstract, Keywords: "Infant, newborn" OR newborn* OR Infant* OR "child, preschool" OR child* OR adolescen* OR pediatric* OR paediatric* OR minor* OR toddler* OR kid OR youth* OR boy OR girl OR schoolchild* OR preschool*
AND
Search W/In ArticleTitle, Abstract, Keywords: "Prescription drugs" OR "nonprescription drugs" OR "controlled substances" OR "pharmaceutical preparations" OR medication* OR medicine* OR pharmaceutical* OR drug* OR prescription* OR Interven* OR prevent* OR reduc* OR "risk reduction behavior" OR "harm reduction" OR educat* OR counsel* OR supervis* OR parenting OR train* OR teach* OR safety OR packag* OR "drug packaging" OR label* OR "drug labeling" OR storage OR "drug storage" OR childproof OR "child resistant" OR "child restraint systems" OR "poison control"
AND
Search W/In All Fields: Afghanistan OR Algeria OR Angola OR Armenia OR Azerbaijan OR Bangladesh OR Belarus OR Belize OR Benin OR Bhutan OR Bolivia OR Bosnia OR Herzegovina OR Bosnia AND Herzegovina OR Brazil OR Bulgaria OR "Burkina Faso" OR Burundi OR "Cabo Verde" OR Cambodia OR Cameroon OR "Central African Republic" OR Chad OR China OR Colombia OR Comoros OR Congo OR "Cote D'ivoire" OR Djibouti OR Egypt OR "El Salvador" OR Guinea OR Eritrea OR ESwatini OR Ethiopia OR Fiji OR Gambia OR Georgia OR Ghana OR Guatemala OR Guyana OR Haiti OR Honduras OR India OR Indonesia OR Iran OR Iraq OR Jamaica OR Jordan OR Kazakhstan OR Kenya OR Kiribati OR Kosovo OR Kyrgyzstan OR Laos OR Lesotho OR Liberia OR Madagascar OR Malawi OR Mali OR "Marshall Islands" OR Mauritania OR Micronesia OR Moldova OR Mongolia OR Morocco OR Mozambique OR Myanmar OR Namibia OR Nepal OR Nicaragua OR Niger OR Nigeria OR "North Macedonia" OR "Republic of North Macedonia" OR Pakistan OR Philippines OR Romania OR Russia OR Rwanda OR Samoa OR Sao Tome OR Senegal OR Sierra Leone OR "Solomon Islands" OR Melanesia OR Somalia OR Sudan OR “Sri Lanka” OR Syria OR Tajikistan OR Tanzania OR Thailand OR Timor-Leste OR Togo OR Tunisia OR Turkey OR Turkmenistan OR Uganda OR Ukraine OR Uzbekistan OR Vanuatu OR Vietnam OR "West Bank" OR Gaza OR "Middle East" OR Yemen OR Zambia OR Zimbabwe OR "Developing countries" OR "Developing countr*" OR "Low income countr*" OR "middle income countr*" OR "low-middle income countr*" OR LMIC OR "third world" OR "resource poor" OR "low resource" OR "under resourced" OR underdevelop*

Web of Science
Abstract poison* OR ingest* OR toxic* OR intoxicat*
AND
Abstract "Infant, newborn" OR newborn* OR Infant* OR "child, preschool" OR preschool OR child* OR adolescen* OR pediatric* OR paediatric* OR minor* OR toddler* OR kid OR youth* OR boy OR girl OR schoolchild*
AND
Abstract "Prescription drugs" OR "nonprescription drugs" OR "controlled substances" OR "pharmaceutical preparations" OR medication* OR medicine* OR pharmaceutical* OR drug* OR prescription* OR Interven* OR prevent* OR reduc* OR "risk reduction behavior" OR "harm reduction" OR educat* OR counsel* OR supervis* OR parenting OR train* OR teach* OR safety OR packag* OR "drug packaging" OR label* OR "drug labeling" OR storage OR "drug storage" OR childproof OR "child resistant" OR "child restraint systems" OR "poison control"
AND
Abstract Afghanistan OR Algeria OR Angola OR Armenia OR Azerbaijan OR Bangladesh OR Belarus OR Belize OR Benin OR Bhutan OR Bolivia OR Bosnia OR Herzegovina OR Bosnia AND Herzegovina OR Brazil OR Bulgaria OR "Burkina Faso" OR Burundi OR "Cabo Verde" OR Cambodia OR Cameroon OR "Central African Republic" OR Chad OR China OR Colombia OR Comoros OR Congo OR "Cote D'Ivoire" OR Djibouti OR Egypt OR "El Salvador" OR Guinea OR Eritrea OR ESwatini OR Ethiopia OR Fiji OR Gambia OR Georgia OR Ghana OR Guatemala OR Guyana OR Haiti OR Honduras OR India OR Indonesia OR Iran OR Iraq OR Jamaica OR Jordan OR Kazakhstan OR Kenya OR Kiribati OR Kosovo OR Kyrgyzstan OR Laos OR Lesotho OR Liberia OR Madagascar OR Malawi OR Mali OR "Marshall Islands" OR Mauritania OR Micronesia OR Moldova OR Mongolia OR Morocco OR Mozambique OR Myanmar OR Namibia OR Nepal OR Nicaragua OR Niger OR Nigeria OR "North Macedonia" OR "Republic of North Macedonia" OR Pakistan OR Philippines OR Romania OR Russia OR Rwanda OR Samoa OR Sao Tome OR Senegal OR Sierra Leone OR "Solomon Islands" OR Melanesia OR Somalia OR Sudan OR “Sri Lanka” OR Syria OR Tajikistan OR Tanzania OR Thailand OR Timor-Leste OR Togo OR Tunisia OR Turkey OR Turkmenistan OR Uganda OR Ukraine OR Uzbekistan OR Vanuatu OR Vietnam OR "West Bank" OR Gaza OR "Middle East" OR Yemen OR Zambia OR Zimbabwe OR "Developing countries" OR "Developing countr*" OR "Low income countr*" OR "middle income countr*" OR "low-middle income countr*" OR LMIC OR "third world" OR "resource poor" OR "low resource" OR "under resourced" OR underdevelop*

CINAHL

((MH Poisoning+) OR (TI poison* OR AB poison*) OR (TI ingest* OR AB ingest*) OR (TI toxic* OR AB toxic*) OR (TI intoxicat* OR AB intoxicat*))

AND

((MH "Infant, newborn+") OR (TI newborn* OR AB newborn*) OR (TI Infant* OR AB Infant*) OR (MH infant+) OR (MH "child, preschool+") OR (TI preschool OR AB preschool) OR (MH child+) OR (TI child* OR AB child*) OR (MH adolescent+) OR (TI adolescen* OR AB adolescen*) OR (TI pediatric* OR AB pediatric*) OR (MH pediatrics+) OR (TI paediatric* OR AB paediatric*) OR (TI minor* OR AB minor*) OR (TI toddler* OR AB toddler*) OR (TI kid OR AB kid) OR (TI youth* OR AB youth*) OR (TI boy OR AB boy) OR (TI girl OR AB girl) OR (TI schoolchild* OR AB schoolchild*) OR (TI preschool* OR AB preschool*))

AND

((MH "Prescription drugs+") OR (MH "nonprescription drugs+") OR (MH "controlled substances+") OR (MH "pharmaceutical preparations+") OR medication* OR medicine* OR pharmaceutical* OR drug* OR prescription* OR Interven* OR prevent* OR (MH "accident prevention+") OR (MH "primary prevention+") OR (MH "secondary prevention+") OR (MH "tertiary prevention+") OR reduc* OR (MH "risk reduction behavior+") OR (MH "harm reduction+") OR educat* OR (MH education+) OR counsel* OR (MH counseling+) OR supervis* OR parenting OR (MH parenting+) OR train* OR teach* OR (MH teaching+) OR safety OR (MH safety+) OR packag* OR (MH "drug packaging+") OR label* OR (MH "drug labeling+") OR storage OR (MH "drug storage+") OR childproof OR "child resistant" OR (MH "child restraint systems+") OR "poison control" OR treatment OR management)

AND

(Afghanistan OR (MH Afghanistan+) OR Algeria OR (MH Algeria+) OR Angola OR (MH Angola+) OR Armenia OR (MH Armenia+) OR Azerbaijan OR (MH Azerbaijan+) OR Bangladesh OR (MH Bangladesh+) OR Belarus OR (MH Belarus+) OR Belize OR (MH Belize+) OR Benin OR (MH Benin+) OR Bhutan OR (MH Bhutan+) OR Bolivia OR (MH Bolivia+) OR Bosnia OR Herzegovina OR Bosnia AND (MH Herzegovina+) OR (MH Brazil+) OR Bulgaria OR (MH Bulgaria+) OR "Burkina Faso" OR (MH "Burkina Faso+") OR Burundi OR (MH Burundi+) OR "Cabo Verde" OR (MH "Cabo Verde+") OR Cambodia OR (MH Cambodia+) OR Cameroon OR (MH Cameroon+) OR "Central African Republic" OR (MH "Central African Republic+") OR Chad OR (MH Chad+) OR China OR (MH China+) OR Colombia OR (MH Colombia+) OR Comoros OR (MH Comoros+) OR Congo OR (MH Congo+) OR "Cote D'Ivoire" OR (MH "Cote D'Ivoire+") OR Djibouti OR (MH Djibouti+) OR Egypt OR (MH Egypt+) OR "El Salvador" OR (MH "El Salvador+") OR Guinea OR (MH Guinea+) OR Eritrea OR (MH Eritrea+) OR ESwatini OR (MH Eswatini+) OR Ethiopia OR (MH Ethiopia+) OR Fiji OR (MH Fiji+) OR Gambia OR (MH Gambia+) OR Georgia OR Georgia (Republic) OR Ghana OR (MH Ghana+) OR Guatemala OR (MH Guatemala+) OR Guyana OR (MH Guyana+) OR Haiti OR (MH Haiti+) OR Honduras OR (MH Honduras+) OR India OR (MH India+) OR Indonesia OR (MH Indonesia+) OR Iran OR (MH Iran+) OR Iraq OR (MH Iraq+) OR Jamaica OR (MH Jamaica+) OR Jordan OR (MH Jordan+) OR Kazakhstan OR (MH Kazakhstan+) OR Kenya OR (MH Kenya+) OR Kiribati OR (MH Kiribati+) OR Kosovo OR (MH Kosovo+) OR Kyrgyzstan OR (MH Kyrgyzstan+) OR Laos OR Lao OR (MH Laos+) OR Lesotho OR (MH Lesotho+) OR Liberia OR (MH Liberia+) OR Madagascar OR (MH Madagascar+) OR Malawi OR (MH Malawi+) OR Mali OR (MH Mali+) OR "Marshall Islands" OR Mauritania OR (MH Mauritania+) OR Micronesia OR (MH Micronesia+) OR Moldova OR (MH Moldova+) OR Mongolia OR (MH Mongolia+) OR Morocco OR (MH Morocco+) OR Mozambique OR (MH Mozambique+) OR Myanmar OR (MH Myanmar+) OR Namibia OR (MH Namibia+) OR Nepal OR (MH Nepal+) OR Nicaragua OR (MH Nicaragua+) OR Niger OR (MH Niger+) OR Nigeria OR (MH Nigeria+) OR "North Macedonia" OR (MH "Republic of North Macedonia+") OR Pakistan OR (MH Pakistan+) OR Philippines OR (MH Philippines+) OR Romania OR (MH Romania+) OR Russia OR (MH Russia+) OR Rwanda OR (MH Rwanda+) OR Samoa OR (MH Samoa+) OR "Sao Tome" OR "Sao Tome" AND (MH Principe+) OR (MH Senegal+) OR "Sierra Leone" OR (MH "Sierra Leone+") OR "Solomon Islands" OR (MH Melanesia+) OR (MH Somalia+) OR Sudan OR (MH Sudan+) OR "Sri Lanka" OR (MH "Sri Lanka+") OR Syria OR (MH Syria+) OR Tajikistan OR (MH Tajikistan+) OR Tanzania OR (MH Tanzania+) OR Thailand OR (MH Thailand+) OR Timor-Leste OR Togo OR (MH Togo+) OR Tunisia OR (MH Tunisia+) OR Turkey OR (MH Turkey+) OR Turkmenistan OR (MH Turkmenistan+) OR Uganda OR (MH Uganda+) OR Ukraine OR (MH Ukraine+) OR Uzbekistan OR (MH Uzbekistan+) OR Vanuatu OR (MH Vanuatu+) OR Vietnam OR (MH Vietnam+) OR "West Bank" OR Gaza OR (MH "Middle East+") OR Yemen OR (MH Yemen+) OR Zambia OR (MH Zambia+) OR Zimbabwe OR (MH Zimbabwe+) OR (MH "Developing countries+") OR "Developing countr*" OR "Low income countr*" OR "middle income countr*" OR "low-middle income countr*" OR LMIC OR "third world" OR "resource poor" OR "low resource" OR "under resourced" OR underdevelop*)

EMBASE
(Poisoning/exp OR poison*:ti,ab OR ingest*:ti,ab OR toxic*:ti,ab OR intoxicat*:ti,ab)
AND
(newborn*:ti,ab OR Infant*:ti,ab OR infant/exp OR preschool:ti,ab OR child/exp OR child*:ti,ab OR adolescent/exp OR adolescen*:ti,ab OR pediatric*:ti,ab OR pediatrics/exp OR paediatric*:ti,ab OR minor*:ti,ab OR toddler*:ti,ab OR kid:ti,ab OR youth*:ti,ab OR boy:ti,ab OR girl:ti,ab OR schoolchild*:ti,ab OR preschool*:ti,ab)
AND
("Prescription drugs"/exp OR "nonprescription drugs"/exp OR "controlled substances"/exp OR "pharmaceutical preparations"/exp OR "accident prevention"/exp OR "primary prevention"/exp OR "secondary prevention"/exp OR "tertiary prevention"/exp OR "risk reduction behavior"/exp OR "harm reduction"/exp OR education/exp OR counseling/exp OR parenting/exp OR "drug packaging"/exp OR teaching/exp OR safety/exp OR "drug labeling"/exp OR "drug storage"/exp OR medication* OR medicine* OR pharmaceutical* OR drug* OR prescription* OR Interven* OR prevent* OR reduc* OR educat* OR counsel* OR supervis* OR parenting OR train* OR teach* OR safety OR packag* OR label* OR storage OR childproof OR "child resistant" OR "poison control" OR treatment OR management)
AND
(Afghanistan OR Afghanistan/exp OR Algeria OR Algeria/exp OR Angola OR Angola/exp OR Armenia OR Armenia/exp OR Azerbaijan OR Azerbaijan/exp OR Bangladesh OR Bangladesh/exp OR Belarus OR Belarus/exp OR Belize OR Belize/exp OR Benin OR Benin/exp OR Bhutan OR Bhutan/exp OR Bolivia OR Bolivia/exp OR Bosnia OR Herzegovina OR Bosnia AND Herzegovina/exp OR Brazil/exp OR Bulgaria OR Bulgaria/exp OR "Burkina Faso" OR "Burkina Faso"/exp OR Burundi OR Burundi/exp OR "Cabo Verde" OR "Cabo Verde"/exp OR Cambodia OR Cambodia/exp OR Cameroon OR Cameroon/exp OR "Central African Republic" OR "Central African Republic"/exp OR Chad OR Chad/exp OR China OR China/exp OR Colombia OR Colombia/exp OR Comoros OR Comoros/exp OR Congo OR Congo/exp OR "Cote D'ivoire" OR "Cote D'ivoire"/exp OR Djibouti OR Djibouti/exp OR Egypt OR Egypt/exp OR "El Salvador" OR "El Salvador"/exp OR Guinea OR Guinea/exp OR Eritrea OR Eritrea/exp OR Eswatini OR Eswatini/exp OR Ethiopia OR Ethiopia/exp OR Fiji OR Fiji/exp OR Gambia OR Gambia/exp OR Georgia OR Ghana OR Ghana/exp OR Guatemala OR Guatemala/exp OR Guyana OR Guyana/exp OR Haiti OR Haiti/exp OR Honduras OR Honduras/exp OR India OR India/exp OR Indonesia OR Indonesia/exp OR Iran OR Iran/exp OR Iraq OR Iraq/exp OR Jamaica OR Jamaica/exp OR Jordan OR Jordan/exp OR Kazakhstan OR Kazakhstan/exp OR Kenya OR Kenya/exp OR Kiribati OR Kiribati/exp OR Kosovo OR Kosovo/exp OR Kyrgyzstan OR Kyrgyzstan/exp OR Laos OR Lao OR Laos/exp OR Lesotho OR Lesotho/exp OR Liberia OR Liberia/exp OR Madagascar OR Madagascar/exp OR Malawi OR Malawi/exp OR Mali OR Mali/exp OR "Marshall Islands" OR Mauritania OR Mauritania/exp OR Micronesia OR Micronesia/exp OR Moldova OR Moldova/exp OR Mongolia OR Mongolia/exp OR Morocco OR Morocco/exp OR Mozambique OR Mozambique/exp OR Myanmar OR Myanmar/exp OR Namibia OR Namibia/exp OR Nepal OR Nepal/exp OR Nicaragua OR Nicaragua/exp OR Niger OR Niger/exp OR Nigeria OR Nigeria/exp OR "North Macedonia" OR "Republic of North Macedonia"/exp OR Pakistan OR Pakistan/exp OR Philippines OR Philippines/exp OR Romania OR Romania/exp OR Russia OR Russia/exp OR Rwanda OR Rwanda/exp OR Samoa OR Samoa/exp OR "Sao Tome" OR "Sao Tome" AND Principe/exp OR Senegal/exp OR "Sierra Leone" OR "Sierra Leone"/exp OR "Solomon Islands" OR Melanesia/exp OR Somalia/exp OR Sudan OR Sudan/exp OR "Sri Lanka" OR "Sri Lanka"/exp OR Syria OR Syria/exp OR Tajikistan OR Tajikistan/exp OR Tanzania OR Tanzania/exp OR Thailand OR Thailand/exp OR Timor-Leste OR Togo OR Togo/exp OR Tunisia OR Tunisia/exp OR Turkey OR Turkey/exp OR Turkmenistan OR Turkmenistan/exp OR Uganda OR Uganda/exp OR Ukraine OR Ukraine/exp OR Uzbekistan OR Uzbekistan/exp OR Vanuatu OR Vanuatu/exp OR Vietnam OR Vietnam/exp OR "West Bank" OR Gaza OR "Middle East"/exp OR Yemen OR Yemen/exp OR Zambia OR Zambia/exp OR Zimbabwe OR Zimbabwe/exp OR "Developing countr*" OR "Low income countr*" OR "middle income countr*" OR "low-middle income countr*" OR LMIC OR "third world" OR "resource poor" OR "low resource" OR "under resourced" OR underdevelop*)

PsycInfo
Abstract poison* OR ingest* OR toxic* OR intoxicat*
AND
Abstract "Infant, newborn" OR newborn* OR Infant* OR "child, preschool" OR preschool OR child* OR adolescen* OR pediatric* OR paediatric* OR minor* OR toddler* OR kid OR youth* OR boy OR girl OR schoolchild*
AND
Full Text "Prescription drugs" OR "nonprescription drugs" OR "controlled substances" OR "pharmaceutical preparations" OR medication* OR medicine* OR pharmaceutical* OR drug* OR prescription* OR Interven* OR prevent* OR reduc* OR "risk reduction behavior" OR "harm reduction" OR educat* OR counsel* OR supervis* OR parenting OR train* OR teach* OR safety OR packag* OR "drug packaging" OR label* OR "drug labeling" OR storage OR "drug storage" OR childproof OR "child resistant" OR "child restraint systems" OR "poison control"

AND
Full Text Afghanistan OR Algeria OR Angola OR Armenia OR Azerbaijan OR Bangladesh OR Belarus OR Belize OR Benin OR Bhutan OR Bolivia OR Bosnia OR Herzegovina OR Brazil OR Bulgaria OR Burkina Faso OR Burundi OR Cabo Verde OR Cambodia OR Cameroon OR Central African Republic OR Chad OR China OR Colombia OR Comoros OR Congo OR Cote Divoire OR Djibouti OR Egypt OR "El Salvador" OR Guinea OR Eritrea OR ESwatini OR Ethiopia OR Fiji OR Gambia OR Georgia OR Ghana OR Guatemala OR Guyana OR Haiti OR Honduras OR India OR Indonesia OR Iran OR Iraq OR Jamaica OR Jordan OR Kazakhstan OR Kenya OR Kiribati OR Kosovo OR Kyrgyzstan OR Laos OR Lesotho OR Liberia OR Madagascar OR Malawi OR Mali OR "Marshall Islands" OR Mauritania OR Micronesia OR Moldova OR Mongolia OR Morocco OR Mozambique OR Myanmar OR Namibia OR Nepal OR Nicaragua OR Niger OR Nigeria OR North Macedonia OR Pakistan OR Philippines OR Romania OR Russia OR Rwanda OR Samoa OR Sao Tome OR Senegal OR Sierra Leone OR Solomon Islands OR Melanesia OR Somalia OR Sudan OR Sri Lanka OR Syria OR Tajikistan OR Tanzania OR Thailand OR Timor Leste OR Togo OR Tunisia OR Turkey OR Turkmenistan OR Uganda OR Ukraine OR Uzbekistan OR Vanuatu OR Vietnam OR West Bank OR Gaza OR Yemen OR Zambia OR Zimbabwe OR "Developing countries" OR "Developing countr*" OR "Low income countr*" OR "middle income countr*" OR "low-middle income countr*" OR LMIC OR "third world" OR "resource poor" OR "low resource" OR "under resourced" OR underdevelop*

Cochrane databases
(poison*:ti,ab OR ingest*:ti,ab OR toxic*:ti,ab OR intoxicat*:ti,ab)
AND
([mh "Infant, newborn"] OR newborn*:ti,ab OR Infant*:ti,ab OR [mh infant] OR [mh "child, preschool"] OR preschool:ti,ab OR [mh child] OR child*:ti,ab OR [mh adolescent] OR adolescen*:ti,ab OR pediatric*:ti,ab OR [mh pediatrics] OR paediatric*:ti,ab OR minor*:ti,ab OR toddler*:ti,ab OR kid:ti,ab OR youth*:ti,ab OR boy:ti,ab OR girl:ti,ab OR schoolchild*:ti,ab OR preschool*:ti,ab)
AND
([mh "Prescription drugs"] OR [mh "nonprescription drugs"] OR [mh "controlled substances"] OR [mh "pharmaceutical preparations"] OR medication* OR medicine* OR pharmaceutical* OR drug* OR prescription* OR Interven* OR prevent* OR [mh "accident prevention"] OR [mh "primary prevention"] OR [mh "secondary prevention"] OR [mh "tertiary prevention"] OR reduc* OR [mh "risk reduction behavior"] OR [mh "harm reduction"] OR educat* OR [mh education] OR counsel* OR [mh counseling] OR supervis* OR parenting OR [mh parenting] OR train* OR teach* OR [mh teaching] OR safety OR [mh safety] OR packag* OR [mh "drug packaging"] OR label* OR [mh "drug labeling"] OR storage OR [mh "drug storage"] OR childproof OR "child resistant" OR [mh "child restraint systems"] OR "poison control" OR treatment OR management)
AND
Afghanistan OR [mh Afghanistan] OR Algeria OR [mh Algeria] OR Angola OR [mh Angola] OR Armenia OR [mh Armenia] OR Azerbaijan OR [mh Azerbaijan] OR Bangladesh OR [mh Bangladesh] OR Belarus OR [mh Belarus] OR Belize OR [mh Belize] OR Benin OR [mh Benin] OR Bhutan OR [mh Bhutan] OR Bolivia OR [mh Bolivia] OR Bosnia OR Herzegovina OR Bosnia AND [mh Herzegovina] OR [mh Brazil] OR Bulgaria OR [mh Bulgaria] OR "Burkina Faso" OR [mh "Burkina Faso"] OR Burundi OR [mh Burundi] OR "Cabo Verde" OR [mh "Cabo Verde"] OR Cambodia OR [mh Cambodia] OR Cameroon OR [mh Cameroon] OR "Central African Republic" OR [mh "Central African Republic"] OR Chad OR [mh Chad] OR China OR [mh China] OR Colombia OR [mh Colombia] OR Comoros OR [mh Comoros] OR Congo OR [mh Congo] OR "Cote D'ivoire" OR [mh "Cote D'ivoire"] OR Djibouti OR [mh Djibouti] OR Egypt OR [mh Egypt] OR "El Salvador" OR [mh "El Salvador"] OR Guinea OR [mh Guinea] OR Eritrea OR [mh Eritrea] OR ESwatini OR [mh Eswatini] OR Ethiopia OR [mh Ethiopia] OR Fiji OR [mh Fiji] OR Gambia OR [mh Gambia] OR Georgia OR Georgia (Republic) OR Ghana OR [mh Ghana] OR Guatemala OR [mh Guatemala] OR Guyana OR [mh Guyana] OR Haiti OR [mh Haiti] OR Honduras OR [mh Honduras] OR India OR [mh India] OR Indonesia OR [mh Indonesia] OR Iran OR [mh Iran] OR Iraq OR [mh Iraq] OR Jamaica OR [mh Jamaica] OR Jordan OR [mh Jordan] OR Kazakhstan OR [mh Kazakhstan] OR Kenya OR [mh Kenya] OR Kiribati OR [mh Kiribati] OR Kosovo OR [mh Kosovo] OR Kyrgyzstan OR [mh Kyrgyzstan] OR Laos OR Lao OR [mh Laos] OR Lesotho OR [mh Lesotho] OR Liberia OR [mh Liberia] OR Madagascar OR [mh Madagascar] OR Malawi OR [mh Malawi] OR Mali OR [mh Mali] OR "Marshall Islands" OR Mauritania OR [mh Mauritania] OR Micronesia OR [mh Micronesia] OR Moldova OR [mh Moldova] OR Mongolia OR [mh Mongolia] OR Morocco OR [mh Morocco] OR Mozambique OR [mh Mozambique] OR Myanmar OR [mh Myanmar] OR Namibia OR [mh Namibia] OR Nepal OR [mh Nepal] OR Nicaragua OR [mh Nicaragua] OR Niger OR [mh Niger] OR Nigeria OR [mh Nigeria] OR "North Macedonia" OR [mh "Republic of North Macedonia"] OR Pakistan OR [mh Pakistan] OR Philippines OR [mh Philippines] OR Romania OR [mh Romania] OR Russia OR [mh Russia] OR Rwanda OR [mh Rwanda] OR Samoa OR [mh Samoa] OR "Sao Tome" OR "Sao Tome" AND [mh Principe] OR [mh Senegal] OR "Sierra Leone" OR [mh "Sierra Leone"] OR "Solomon Islands" OR [mh Melanesia] OR [mh Somalia] OR Sudan OR [mh Sudan] OR "Sri Lanka" OR [mh "Sri Lanka"] OR Syria OR [mh Syria] OR Tajikistan OR [mh Tajikistan] OR Tanzania OR [mh Tanzania] OR Thailand OR [mh Thailand] OR Timor-Leste OR Togo OR [mh Togo] OR Tunisia OR [mh Tunisia]

OR Turkey OR [mh Turkey] OR Turkmenistan OR [mh Turkmenistan] OR Uganda OR [mh Uganda] OR Ukraine OR [mh Ukraine] OR Uzbekistan OR [mh Uzbekistan] OR Vanuatu OR [mh Vanuatu] OR Vietnam OR [mh Vietnam] OR "West Bank" OR Gaza OR [mh "Middle East"] OR Yemen OR [mh Yemen] OR Zambia OR [mh Zambia] OR Zimbabwe OR [mh Zimbabwe] OR [mh "Developing countries"] OR (Developing countr*) OR (low income countr*) OR (middle income countr*) OR (low-middle income countr*) OR LMIC OR third world OR resource poor OR low resource OR under resourced OR underdevelop*

Global Index Medicus
Title/Abstract poison* OR ingest* OR toxic* OR toxin* OR intoxicat*
AND
Title/Abstract "Infant, newborn" OR newborn* OR Infant* OR "child, preschool" OR preschool OR child* OR adolescen* OR pediatric* OR paediatric* OR minor* OR toddler* OR kid OR youth* OR boy OR girl OR schoolchild*
AND
Title/Abstract "Prescription drugs" OR "nonprescription drugs" OR "controlled substances" OR "pharmaceutical preparations" OR medication* OR medicine* OR pharmaceutical* OR drug* OR prescription* OR Interven* OR prevent* OR reduc* OR "risk reduction behavior" OR "harm reduction" OR educat* OR counsel* OR supervis* OR parenting OR train* OR teach* OR safety OR packag* OR "drug packaging" OR label* OR "drug labeling" OR storage OR "drug storage" OR childproof OR "child resistant" OR "child restraint systems" OR "poison control"
AND
Title/Abstract Afghanistan OR Algeria OR Angola OR Armenia OR Azerbaijan OR Bangladesh OR Belarus OR Belize OR Benin OR Bhutan OR Bolivia OR Bosnia OR Herzegovina OR Brazil OR Bulgaria OR "Burkina Faso" OR Burundi OR "Cabo Verde" OR Cambodia OR Cameroon OR "Central African Republic" OR Chad OR China OR Colombia OR Comoros OR Congo OR "Cote D'ivoire" OR Djibouti OR Egypt OR "El Salvador" OR Guinea OR Eritrea OR Eswatini OR Ethiopia OR Fiji OR Gambia OR Georgia OR Ghana OR Guatemala OR Guyana OR Haiti OR Honduras OR India OR Indonesia OR Iran OR Iraq OR Jamaica OR Jordan OR Kazakhstan OR Kenya OR Kiribati OR Kosovo OR Kyrgyzstan OR Laos OR Lesotho OR Liberia OR Madagascar OR Malawi OR Mali OR "Marshall Islands" OR Mauritania OR Micronesia OR Moldova OR Mongolia OR Morocco OR Mozambique OR Myanmar OR Namibia OR Nepal OR Nicaragua OR Niger OR Nigeria OR "North Macedonia" OR "Republic of North Macedonia" OR Pakistan OR Philippines OR Romania OR Russia OR Rwanda OR Samoa OR Sao Tome OR Senegal OR Sierra Leone OR "Solomon Islands" OR Melanesia OR Somalia OR Sudan OR “Sri Lanka” OR Syria OR Tajikistan OR Tanzania OR Thailand OR Timor-Leste OR Togo OR Tunisia OR Turkey OR Turkmenistan OR Uganda OR Ukraine OR Uzbekistan OR Vanuatu OR Vietnam OR "West Bank" OR Gaza OR "Middle East" OR Yemen OR Zambia OR Zimbabwe OR "Developing countries" OR "Developing countr*" OR "Low income countr*" OR "middle income countr*" OR "low-middle income countr*" OR LMIC OR "third world" OR "resource poor" OR "low resource" OR "under resourced" OR underdevelop*

Appendix 2: Critical appraisal of studies

Quasi-Experimental Critical Appraisal Checklist

	Foulds 2021	Justin 2014	Mandiracioglu 2003	Mohamad 2018	Das 2020	Wafik 2014
Is it clear in the study what is the 'cause' and what is the 'effect'?	Yes	Yes	Yes	Yes	Yes	Yes
Were the participants included in any comparisons similar?	Yes	Yes	Yes	Yes	Yes	Yes
Were the participants included in any comparisons receiving similar treatment/care, other than the exposure or intervention of interest?	N/A	N/A	N/A	N/A	N/A	N/A
Was there a control group?	No	No	No	No	No	No
Were there multiple measurements of the outcome both pre and post the intervention/exposure?	No	Yes	No	No	No	Yes
Was follow up complete and if not, were differences between groups in terms of their follow up adequately described and analyzed?	Yes	Yes	Yes	Yes	Yes	Yes
Were the outcomes of participants included in any comparisons measured in the same way?	Yes	Yes	Yes	Yes	Yes	Yes
Were outcomes measured in a reliable way?	Yes	Yes	Yes	Yes	Yes	Yes
Was appropriate statistical analysis used?	Yes	Yes	Yes	Yes	Yes	Yes
**Score**	6	7	6	6	6	7

Randomized Controlled Trial Critical Appraisal Checklist

	Rehmani 2010
Was true randomization used for assignment of participants to treatment groups?	Yes
Was allocation to treatment groups concealed?	Yes
Were treatment groups similar at the baseline?	Yes
Were participants blind to treatment assignment?	No
Were those delivering treatment blind to treatment assignment?	No
Were outcomes assessors blind to treatment assignment?	Unclear
Were treatment groups treated identically other than the intervention of interest?	No
Was follow up complete and if not, were differences between groups in terms of their follow up adequately described and analyzed?	Yes
Were participants analyzed in the groups to which they were randomized?	Yes
Were outcomes measured in the same way for treatment groups?	Yes
Were outcomes measured in a reliable way?	Yes
Was appropriate statistical analysis used?	Yes
Was the trial design appropriate, and any deviations from the standard RCT design (individual randomization, parallel groups) accounted for in the conduct and analysis of the trial?	Yes
**Score**	9

Case Series Critical Appraisal Checklist

	Bek 2008	Eddleston 2007	Musumba 2004	Ozdemir 2011	Singhi 2003
Were there clear criteria for inclusion in the case series?	Yes	Yes	Unclear	Yes	Yes
Was the condition measured in a standard, reliable way for all participants included in the case series?	Yes	Yes	Yes	Yes	Yes
Were valid methods used for identification of the condition for all participants included in the case series?	Yes	Yes	Yes	No	No
Did the case series have consecutive inclusion of participants?	Yes	Yes	Unclear	Yes	Yes
Did the case series have complete inclusion of participants?	Yes	Yes	Unclear	Yes	Yes
Was there clear reporting of the demographics of the participants in the study?	Yes	No	No	No	Yes
Was there clear reporting of clinical information of the participants?	Yes	Yes	Yes	Yes	No
Were the outcomes or follow up results of cases clearly reported?	No	Yes	Yes	Yes	No
Was there clear reporting of the presenting site(s)/clinic(s) demographic information?	No	No	No	No	No
Was statistical analysis appropriate?	N/A	N/A	N/A	N/A	N/A
**Score**	7	7	4	6	5

Case-Control Critical Appraisal Checklist*

	Ahmed 2011a	Ahmed 2011b	Chatsantiprapa 2001	Dayasiri 2017	Haidar 2020	Ramos 2010	Soori 2001
Were the groups comparable other than the presence of disease in cases or the absence of disease in controls?	Yes	Yes	Yes	Yes	Yes	Yes	Yes
Were cases and controls matched appropriately?	Yes	Yes	Yes	Yes	Yes	Yes	Yes
Were the same criteria used for identification of cases and controls?	Yes	Yes	Yes	Yes	Yes	Yes	Yes
Was exposure measured in a standard, valid and reliable way?	No	No	No	Yes	Yes	No	No
Was exposure measured in the same way for cases and controls?	Yes	Yes	Yes	Yes	No	Yes	Yes
Were confounding factors identified?	Yes	Yes	No	Yes	Yes	Yes	No
Were strategies to deal with confounding factors stated?	Yes	Yes	No	Yes	Yes	Yes	No
Were outcomes assessed in a standard, valid and reliable way for cases and controls?	Yes	Yes	Yes	Yes	Yes	Yes	Yes
Was the exposure period of interest long enough to be meaningful?	Unclear	Unclear	Unclear	Unclear	Unclear	Unclear	Unclear
Was appropriate statistical analysis used?	Yes	Yes	Yes	Yes	Yes	Yes	Yes
**Score**	8	8	6	9	8	8	6

*All studies pertain to the epidemiology or risk factors associated with poisoning due to pharmaceuticals

Descriptive Cross-Sectional Critical Appraisal Checklist*

Author, Year	Was the sample frame appropriate to address the target population?	Were study participants sampled in an appropriate way?	Was the sample size adequate?	Were the study subjects and the setting described in detail?	Was the data analysis conducted with sufficient coverage of the identified sample?	Were valid methods used for the identification of the condition?	Was the condition measured in a standard, reliable way for all participants?	Was appropriate statistical analysis used?	Was the response rate adequate?	Score
Adejuyigbe 2002	No	Yes	No	Yes	Yes	No	Yes	Yes	Yes	6
Aggarwal 2014	No	No	No	No	Yes	No	Yes	Yes	Yes	4
Ahmed 2020	No	Yes	No	No	Yes	No	Yes	Yes	Yes	5
Andiran 2004	No	Yes	Yes	No	No	No	Yes	Yes	Yes	5
Ansong 2016	No	Yes	Yes	No	Yes	No	Yes	Yes	Yes	6
Arslan 2007	Yes	Yes	No	No	Yes	Yes	Yes	Yes	Yes	7
Azab 2016	Yes	Yes	Yes	Yes	Yes	No	Yes	Yes	Yes	8
Aziz 2016	No	Yes	No	No	No	No	Yes	Yes	Yes	4
Bacha 2015	Yes	Yes	No	No	Yes	No	Yes	Yes	Yes	6
Bandyopadh-yay 2017	No	Yes	No	No	Yes	No	Yes	Yes	Yes	5
Bazmamoun 2014	No	Yes	N/A	No	Yes	Yes	Yes	Yes	Yes	6
Benabdellah 2020	No	Yes	No	No	Yes	No	Yes	Yes	Yes	5
Boyle 2020	No	No	No	Yes	Yes	No	Yes	Yes	Yes	5
Brata 2013	No	Yes	No	No	Yes	No	Yes	Yes	Yes	5
Budhathoki 2009	No	Yes	No	No	Yes	No	Yes	Yes	Yes	5
Dayachand 2015	No	Yes	No	No	Yes	No	Yes	Yes	Yes	5
Dayasiri 2020	Yes	Yes	Yes	Yes	Yes	No	Yes	Yes	Yes	8
Dayasiri 2018a	No	Yes	Yes	Yes	Yes	No	Yes	Yes	Yes	7
Dayasiri 2018b	Yes	Yes	Yes	Yes	Yes	No	Yes	Yes	Yes	8

Descriptive Cross-Sectional Critical Appraisal Checklist* (continued)

Author	Was the sample frame appropriate to address the target population?	Were study participants sampled in an appropriate way?	Was the sample size adequate?	Were the study subjects and the setting described in detail?	Was the data analysis conducted with sufficient coverage of the identified sample?	Were valid methods used for the identification of the condition?	Was the condition measured in a standard, reliable way for all participants?	Was appropriate statistical analysis used?	Was the response rate adequate?	Score
Dhakal 2014	No	Yes	No	No	Yes	No	Yes	Yes	Yes	5
Diallo 2021	Yes	Yes	Yes	No	Yes	No	Yes	Yes	Yes	7
Edelu 2016	No	Yes	No	Yes	Yes	No	Yes	Yes	Yes	6
El-Tal 2006	No	Yes	No	No	Yes	No	Yes	Yes	Yes	5
Elcioglu 2004	No	No	No	Yes	Yes	No	Yes	Yes	Yes	5
Erkal 2006	No	Yes	No	No	Yes	No	Yes	Yes	Yes	5
Farag 2021	Yes	Yes	Yes	Yes	Yes	No	Yes	Yes	Yes	8
Gheshlaghi 2013	Yes	Yes	Yes	No	Yes	No	Yes	Yes	Yes	7
Gholami 2022	Yes	Yes	No	Yes	Yes	Yes	Yes	Yes	Yes	8
Gupta 2003	Yes	Yes	Yes	No	Yes	No	Yes	Yes	Yes	7
Hamid 2005	No	Yes	Yes	Yes	Yes	No	Yes	Yes	Yes	7
Hyder 2009	No	Yes	No	No	Yes	No	Yes	Yes	Yes	5
Elshoura 2016	No	Yes	No	No	Yes	No	Yes	Yes	Yes	5
Ikhile 2019	No	Yes	No	No	Yes	No	Yes	Yes	Yes	5
Imoudu 2018	No	Yes	No	No	Yes	No	Yes	Yes	Yes	5
Isaac 2022	No	Yes	No	Yes	Yes	No	Yes	Yes	Yes	6
Jayachandran 2016	No	Yes	No	Yes	No	Yes	Yes	Yes	Yes	6
Jayakrishnan 2021	No	Yes	Yes	No	Yes	No	Yes	Yes	Yes	6
Jayashree 2011	No	Yes	No	No	Yes	No	Yes	Yes	Yes	5
Jose 2012	No	Yes	No	No	Yes	No	Yes	Yes	Yes	5

**Descriptive Cross-Sectional Critical Appraisal Checklist*** (continued)										
**Author**	Was the sample frame appropriate to address the target population?	Were study participants sampled in an appropriate way?	Was the sample size adequate?	Were the study subjects and the setting described in detail?	Was the data analysis conducted with sufficient coverage of the identified sample?	Were valid methods used for the identification of the condition?	Was the condition measured in a standard, reliable way for all participants?	Was appropriate statistical analysis used?	Was the response rate adequate?	Score
Dayasiri 2017	No	Yes	No	Yes	Yes	No	Yes	Yes	Yes	6
Khajeh 2012	No	Yes	No	No	Yes	No	Yes	Yes	Yes	5
Khan 2016	No	Yes	No	No	Yes	No	Yes	Yes	Yes	5
Kohli 2008	No	Yes	No	Yes	Yes	No	Yes	Yes	Yes	6
Kumar 2013	No	Yes	No	No	Yes	No	Yes	Yes	Yes	5
Kurtoglu 2000	No	No	No	No	Yes	No	Yes	Yes	Yes	4
Limjindaporn 2010	No	Yes	No	Yes	Yes	No	Yes	Yes	Yes	6
Lucas 2006	Yes	Yes	Yes	No	Yes	No	Yes	Yes	Yes	7
Maior 2020	Yes	Yes	Yes	Yes	Yes	No	Yes	Yes	Yes	8
Malangu 2014	No	Unclear	No	No	Unclear	No	Yes	Yes	Yes	3
Malla 2011	No	Yes	No	No	Yes	No	Yes	Yes	Yes	5
Manzar 2010	No	Yes	Unclear	No	Yes	No	Yes	Yes	Yes	5
Mehrpour 2015	No	Yes	No	Yes	Yes	No	Yes	Yes	Yes	6
Mekaoui 2016	No	Yes	No	No	Yes	No	Yes	Yes	Yes	5
Mert 2006	No	Yes	No	No	Yes	No	Yes	Yes	Yes	5
Mishra 2017	No	Yes	Yes	No	Yes	No	Yes	Yes	Yes	6
Mishra 2014	No	Yes	No	No	Yes	No	Yes	Yes	Yes	5
Moghadamnia 2004	No	Yes	Unclear	No	Yes	No	Yes	No	Yes	4

Mutiso 2014	No	Yes	No	Yes	Yes	No	Yes	Yes	Yes	6

Descriptive Cross-Sectional Critical Appraisal Checklist* (continued)

**Author**	Was the sample frame appropriate to address the target population?	Were study participants sampled in an appropriate way?	Was the sample size adequate?	Were the study subjects and the setting described in detail?	Was the data analysis conducted with sufficient coverage of the identified sample?	Were valid methods used for the identification of the condition?	Was the condition measured in a standard, reliable way for all participants?	Was appropriate statistical analysis used?	Was the response rate adequate?	Score
Mutlu 2010	No	Yes	Yes	No	Yes	No	Yes	Yes	Yes	6
Narendra 2017	No	Yes	No	No	Yes	No	Yes	Yes	Yes	5
Nguefack 2017	No	Yes	No	Yes	Yes	No	Yes	Yes	Yes	6
Obu 2020	No	Yes	No	Yes	Yes	No	Yes	Yes	Yes	6
Oguche 2007	No	Yes	No	Yes	Yes	No	Yes	Yes	Yes	6
Olatunya 2015	No	Yes	No	Yes	Yes	No	Yes	Yes	Yes	6
Oprescu 2012	No	Yes	No	No	Yes	No	Yes	Yes	Yes	5
Oray 2008	No	Yes	Unclear	No	Yes	No	Yes	Yes	Yes	5
Osaghae 2013	No	Yes	No	No	Yes	No	Yes	Yes	Yes	5
Ozdemir 2012	No	Yes	Yes	No	Yes	No	Yes	Yes	Yes	6
Ozdogan 2008	No	Yes	No	Yes	Yes	No	Yes	Yes	Yes	6
Paudyal 2005	No	Yes	No	Yes	Yes	No	Yes	Yes	Yes	6
Prasadi 2018	Yes	Yes	Yes	Yes	Yes	No	Yes	Yes	Yes	8
Ram 2014	No	Yes	No	No	Yes	No	Yes	Yes	Yes	5
Raman 2015	No	Yes	No	Yes	Yes	No	Yes	Yes	Yes	6
Ramanayake 2012	Yes	Yes	Unclear	Yes	Yes	No	Yes	Yes	Yes	7
Ramawat 2021	No	Yes	No	Yes	Yes	No	Yes	Yes	Yes	6
Rashid 2007	No	Yes	No	No	Yes	No	Yes	Yes	Yes	5
Renu 2017	Yes	Yes	Yes	Yes	Yes	No	Yes	Yes	Yes	8
Roy 2017	No	Yes	No	No	Yes	No	Yes	Yes	Yes	5
Sadeghi-Bojd 2014	No	Yes	No	No	Yes	No	Yes	Yes	Yes	5

Satish 2016	No	Yes	No	No	Yes	No	Yes	Yes	Yes	5
Sawalha 2012	No	Yes	No	Yes	Yes	No	Yes	Yes	Yes	6
Senarathna 2012	Yes	Yes	Yes	No	Yes	No	Yes	Yes	Yes	7
**Descriptive Cross-Sectional Critical Appraisal Checklist*** (continued)										
**Author**	Was the sample frame appropriate to address the target population?	Were study participants sampled in an appropriate way?	Was the sample size adequate?	Were the study subjects and the setting described in detail?	Was the data analysis conducted with sufficient coverage of the identified sample?	Were valid methods used for the identification of the condition?	Was the condition measured in a standard, reliable way for all participants?	Was appropriate statistical analysis used?	Was the response rate adequate?	Score
Shadnia 2013	Yes	Yes	Yes	No	Yes	Yes	Yes	Yes	Yes	8
Sharif 2015	No	Yes	No	Yes	Yes	No	Yes	Yes	Yes	6
Sheikh 2015	No	Yes	No	No	Yes	No	Yes	Yes	Yes	5
Sheriff 2011	No	Yes	No	Yes	Yes	No	Yes	Yes	Unclear	5
Shotar 2005	No	Yes	No	No	Yes	No	Yes	Yes	Yes	5
Shwe 2013	No	Yes	No	Yes	Yes	No	Yes	Yes	Yes	6
Sil 2016	No	Yes	Yes	Yes	Yes	No	Yes	Yes	Yes	7
Suting 2021	No	Yes	No	No	Yes	No	Yes	Yes	Yes	5
Tabrizi 2021	No	Yes	No	Yes	Yes	No	Yes	Yes	Yes	6
Tarvadi 2013	No	Yes	No	No	Yes	No	Yes	Yes	Yes	5
Tirolla 2021	Yes	Yes	No	No	Yes	No	Yes	Yes	Yes	6
Trangadia 2016	No	Yes	No	No	Yes	No	Yes	Yes	Yes	5
Traore 2018	No	Yes	Yes	Yes	Yes	No	Yes	Yes	Yes	7
Ugwu 2012	No	Yes	No	Yes	Yes	No	Yes	Yes	Yes	6
Ulmeanu 2005	No	Yes	No	No	Yes	No	Yes	Yes	Yes	5
Wananukul 2007	Yes	Yes	Yes	No	Yes	No	Yes	Yes	Yes	7
Wang 2017	Yes	Yes	Yes	No	Yes	No	Yes	Yes	Yes	7
Wasim 2021	No	Yes	No	No	Yes	No	Yes	Yes	Yes	5
Winston 2017	No	Yes	Yes	Yes	Yes	No	Yes	Yes	Yes	7
Yasmeen 2010	No	Yes	No	No	Yes	No	Yes	Yes	Yes	5
Z’gambo 2016	No	Yes	Yes	Yes	Yes	No	Yes	Yes	Yes	7
Zarif 2018	No	Yes	Yes	No	Yes	No	Yes	Yes	Yes	6

*All studies pertain to the epidemiology or risk factors associated with poisoning due to pharmaceuticals

Appendix 3: Studies excluded through critical appraisal

Adejuyigbe, E. A., Onayade, A. A., Senbanjo, I. O., & Oseni, S. E. (2002). Childhood poisoning at the Obafemi Awolowo University teaching hospital, Ile-Ife, Nigeria. *Nigerian Journal of Medicine : Journal of the National Association of Resident Doctors of Nigeria, 11*(4), 183-186.

Aggarwal, B., Rana, S. K., & Chhavi, N. (2014). Pattern of poisoning in children, an experience from a teaching hospital in northern India. *JK Science, 16*(4), 174-178. Retrieved from http://www.jkscience.org/archives/volume164/Original%20Article6.pdf

Ahmed, P. A., Nwatah, V. E., & Ulonnam, C. C. (2020). Childhood accidental poisoning among hospitalised children in a tertiary health care in north central Nigeria-A two year prospective report. *Nigerian Journal of Paediatrics, 47*(3)

Andiran, N., & Sarikayalar, F. (2004). Pattern of acute poisonings in childhood in Ankara: What has changed in twenty years? *Turk J Pediatr, 46*(2), 147-52.

Ansong, D., Nkyi, C., Appiah, C. O., Amuzu, E. X., Frimpong, C. A., Nyanor, I., . . . Sylverken, J. (2016). Epidemiology of paediatric poisoning reporting to a tertiary hospital in Ghana. *SAJCH South African Journal of Child Health, 10*(1), 68-70. doi:http://dx.doi.org/10.7196/SAJCH.2016.v10i1.1055

Aziz, U. B. A., Aslami, A. N., & Ali, S. M. (2016). Profile of acute paediatric poisoning cases admitted in a tertiary care centre in north India. *Indian Journal of Forensic Medicine and Toxicology, 10*(1), 217-222. doi:http://dx.doi.org/10.5958/0973-9130.2016.00050.5

Bacha, T., & Tilahun, B. (2015). A cross-sectional study of children with acute poisoning: A three-year retrospective analysis. *World Journal of Emergency Medicine, 6*(4), 265-269. doi:http://dx.doi.org/10.5847/wjem.j.1920-8642.2015.04.003

Bandyopadhyay, A., & Mandal, P. K. (2017). Clinical profile and outcome of acute poisoning in children and adolescent in a tertiary care center. *Asian Journal of Medical Sciences, 8*(3), 76-80.

Bazamamoun, H., Fayyazi, A., Khajeh, A., Sabzee, M. K., & Khezrian, F. (2014). A study of methadone-poisoned children referred to Hamadan's Be’sat Hospital/Iran. *Iranian Journal of*

Child Neurology, 8(2), 34-37. Retrieved from http://proxygw.wrlc.org/login?url=https://search.ebscohost.com/login.aspx?direct=true&db=ccm&AN=100459418&site=ehost-live&scope=site&custid=s8987071

Benabdellah, F. Z., Soulaymani, A., Mokhtari, A., Soulaymani-Bencheikh, R., Khadmaoui, A., & Hami, H. (2020). Economic evaluation of the direct cost resulting from childhood poisoning in Morocco: Micro-costing analysis. *Archives of Public Health, 78*(1) doi:10.1186/s13690-020-00440-z

Boyle, K. L., Periyanayagam, U., Babu, K. M., Rice, B. T., & Bisanzo, M. (2020). Pediatric poisonings in a rural Ugandan emergency department. *Pediatric Emergency Care, 36*(3), E160-E162. doi:http://dx.doi.org/10.1097/PEC.0000000000001265

Brata Ghosh, V., Jhamb, U., Singhal, R., & Krishnan, R. (2013). Common childhood poisonings and their outcome in a tertiary care center in Delhi. *Indian Journal of Pediatrics, 80*(6), 516-518. doi:http://dx.doi.org/10.1007/s12098-012-0879-5

Budhathoki, S., Poudel, P., Shah, D., Bhatta, N. K., Dutta, A. K., Shah, G. S., . . . Singh, M. K. (2009). Clinical profile and outcome of children presenting with poisoning or intoxication: A hospital based study. *Nepal Med Coll J, 11*(3), 170-5.

Chatsantiprapa, K., Chokkanapitak, J., & Pinpradit, N. (2001). Host and environment factors for exposure to poisons: A case-control study of preschool children in Thailand. *Injury Prevention : Journal of the International Society for Child and Adolescent Injury Prevention*, *7*(3), 214-217.

Dayachand, & Kumar, H. (2015). A study of pattern of poisoning among children in NCR, region India. *Medico-Legal Update, 15*(2), 143-145. doi:10.5958/0974-1283.2015.00064.X

Dhakal, A. K., Shrestha, D., Shakya, A., Shah, S. C., & Shakya, H. (2014). Clinical profile of acute poisoning in children at a teaching hospital in Lalitpur. *Journal of Nepal Paediatric Society, 34*(2), 100-103. doi:http://dx.doi.org/10.3126/jnps.v34i2.10139

Edelu, B. O., Odetunde, O. I., Eke, C. B., Uwaezuoke, N. A., & Oguonu, T. (2016). Accidental childhood poisoning in Enugu; South-East; Nigeria. *Ann.Med.Health Sci.Res.(Online), 6*(3), 168-

171. Retrieved from https://www.ajol.info/index.php/amhsr/article/viewFile/139272/128969http://www.amhsr.org/temp/AnnMedHealthSciRes63168-1467901_040439.pdf

Elcioglu, O., Aksoy, S., & Gunduz, T. (2004). Children's rights and a sample study on accidents in children groups aged 0-5 years old in the light of parents' responsibility in Turkey. *Saudi Medical Journal, 25*(4), 470-473.

El-Tal, Y. M., Nayf, M. R., Sameer, M. M., Hussein, A. S., & Khalid, R. O. (2006). Accidental drug poisoning. *Bahrain Medical Bulletin, 28*(2), 69-71.

Erkal, S., & Safak, S. (2006). An evaluation of the poisoning accidents encountered in children aged 0-6 years in Kirikkale. *Turk J Pediatr, 48*(4), 294-300.

Hyder, A. A., Sugerman, D. E., Puvanachandra, P., Razzak, J., El-Sayed, H., Isaza, A., . . . Peden, M. (2009). Global childhood unintentional injury surveillance in four cities in developing countries: A pilot study. *Bulletin of the World Health Organization, 87*(5), 345-352. doi:http://dx.doi.org/10.2471/BLT.08.055798

IA Elshoura, A., M Sherif, M., M Noor El-Deen, T., A Ali, M., A Abbod, M., & A Ghanem, M. (2016). Assessment of acute poisoning among children in Damietta Governorate. *Al-Azhar Medical Journal, 45*(3), 631-644.

Ikhile, I., Chijioke-Nwauche, I., & Orisakwe, O. E. (2019). Childhood drug and non-drug poisoning in Nigeria: An economic appraisal. *Annals of Global Health, 85*(1), 100. doi:http://dx.doi.org/10.5334/aogh.2554

Imoudu, A. I., Afegbua, D. S., Elike, M., Ishola, I., & Abubakar, A. (2018). Acute childhood poisoning in Azare North Eastern Nigeria. *J Adv Med Med Res.2018; 26 (3): 1, 8*

Isaac, W. E., Iliya, J., Adamu, S., Apllos, D., & Oyeniyi, C. (2022). Spectrum of poisoning and outcome among children in a tertiary hospital, North-East Nigeria: A 20 years retrospective review, 2000-2019. *Open Journal of Pediatrics, 12*(1), 100-124.

Jayachandran, M., Chief, T., Richard, G. M., Askar, S. I., & Gope, R. (2016). A study on contemporary trends of acute fatal poisoning of pediatric age group in rural tertiary care hospital. *Medico-Legal Update*, *16*(2), 86-90. doi:http://dx.doi.org/10.5958/0974-1283.2016.00065.7

Jayakrishnan, M. P., Krishnakumar, P., Geeta, M. G., & George, B. (2021). Changing trends of accidental poisoning in children over the last two decades. *Indian Journal of Community Medicine, 46*(2), 350-351. doi:10.4103/ijcm.IJCM_723_20

Jayashree, M., & Singhi, S. (2011). Changing trends and predictors of outcome in patients with acute poisoning admitted to the intensive care. *Journal of Tropical Pediatrics, 57*(5), 340-346. doi:http://dx.doi.org/10.1093/tropej/fmq099

Jose, A., Sivanandam, S., & Matthai, J. (2012). Poisoning in children from an educationally and economically advanced urban area of south India. *Asian Journal of Epidemiology, 5*(4), 123-129. doi:http://dx.doi.org/10.3923/aje.2012.123.129

Kavinda Chandimal Dayasiri, M. B., Jayamanne, S. F., & Jayasinghe, C. Y. (2017). Non-accidental poisoning among children in rural Sri Lanka: A two-year cross sectional study. *Asia Pacific Journal of Medical Toxicology, 6*(4), 109-114. Retrieved from http://apjmt.mums.ac.ir/article_10598_b5960c599b20fb7e6f48f0b2afa312e3.pdf

Khajeh, A., Narouie, B., Noori, N. M., Emamdadi, A., Ghasemi Rad, M., Kaykha, M., & Hanafi-Bojd, H. (2012). Patterns of acute poisoning in childhood and relative factors in Zahedan, southeast Iran. *Shiraz E Medical Journal, 13*(1), 19-27. Retrieved from http://semj.sums.ac.ir/vol13/jan2012/90003.pdf

Khan, N. U., Khan, U. R., Feroze, A., Khan, S. A., Ali, N., Ejaz, K., . . . Fayyaz, J. (2016). Trends of acute poisoning: 22 years' experience from a tertiary care hospital in Karachi, Pakistan. *Journal of the Pakistan Medical Association, 66*(10), 1237-1242. Retrieved from http://jpma.org.pk/PdfDownload/7926.pdf

Kohli, U., Kuttiat, V. S., Lodha, R., & Kabra, S. K. (2008). Profile of childhood poisoning at a tertiary care centre in north India. *Indian Journal of Pediatrics, 75*(8), 791-794. doi:http://dx.doi.org/10.1007/s12098-008-0105-7

Kumar, N., Gupta, A. K., Najotra, D. K., Digra, S. K., Gupta, S., Gupta, P. K., & Kumar, D. (2013). Incidence and pattern of acute poisoning among children aged 1 month to 18 years: An experience from Jammu. *Indian Journal of Forensic Medicine and Toxicology*, *7*(2), 135-138. doi:http://dx.doi.org/10.5958/j.0973-9130.7.2.030

Kurtoglu, S., Caksen, H., & Poyrazoglu, M. H. (2000). Neonatal poisonings in middle Anatolia of Turkey: An analysis of 72 cases. *Journal of Toxicological Sciences, 25*(2), 115-119. doi:http://dx.doi.org/10.2131/jts.25.115

Limjindaporn, C. (2010). Acute poison exposure in the emergency department: A 2-year study in a university hospital. *Journal of the Medical Association of Thailand = Chotmaihet Thangphaet, 93 Suppl 7*, 41.

Malangu, N. (2014). Contribution of plants and traditional medicines to the disparities and similarities in acute poisoning incidents in Botswana, South Africa and Uganda. *African Journal of Traditional, Complementary, and Alternative Medicines : AJTCAM / African Networks on Ethnomedicines, 11*(2), 425-438. doi:http://dx.doi.org/10.4314/ajtcam.v11i2.29

Malla, T., Malla, K. K., Rao, K. S., Gauchan, E., Basnet, S., & Koirala, D. P. (2011). A scenario of poisoning in children in Manipal teaching hospital. *Journal of Nepal Paediatric Society, 31*(2), 83-88. doi:http://dx.doi.org/10.3126/jnps.v31i2.3634

Manzar, N., Saad, S. M. A., Manzar, B., & Fatima, S. S. (2010). The study of etiological and demographic characteristics of acute household accidental poisoning in children - a consecutive case series study from Pakistan. *BMC Pediatrics, 10*, 28. doi:http://dx.doi.org/10.1186/1471-2431-10-28

Mehrpour, O., Sharifi, M. D., & Ebrahimi, M. (2015). Pattern of acute pediatric poisonings in Birjand city, east of Iran. *International Journal of Medical Toxicology and Forensic Medicine, 5*(4), 192-200. Retrieved from http://journals.sbmu.ac.ir/ijmtfm/article/download/IJMTFM-9216/pdf-4

Mekaoui, N., Karboubi, L., Ouadghiri, F. Z., & Dakhama, B. S. B. (2016). Epidemiological aspects of suicide attempts among Moroccan children. *Pan African Medical Journal, 24*, 112. doi:http://dx.doi.org/10.11604/pamj.2016.24.112.7805

Mert, E., & Bilgin, N. G. (2006). Demographical, aetiological and clinical characteristics of poisonings in Mersin, Turkey. *Human and Experimental Toxicology, 25*(4), 217-223. doi:http://dx.doi.org/10.1191/0960327106ht612oa

Mishra, R., Maheshwari, M., & Chanchlani, R. (2014). A study of acute poisoning in children: Three years experience at a tertiary care hospital of central India . *Journal of Evolution of Medical and Dental Sciences, 3*(24), 6669-6675.

Mishra, S., Ramkumar, T. V., Biswas, A. K., & Panigrahi, S. (2017). Childhood poisoning, a rising epidemic in developing nations: Large single centre study. *Journal of Nepal Paediatric Society, 37*(2), 117-121. doi:http://dx.doi.org/10.3126/jnps.v37i2.16843

Moghadamnia, A. -., Esmaeilnia-Shirvani, T., Esmaeili, M. -., Bayati, Z., & Gholitabar, Z. -. (2004). A report of childhood poisoning in Babol. *Archives of Iranian Medicine, 7*(4), 297-299.

Mutiso, V. M., Muoki, A. S., & Kimeu, M. M. (2014). Patterns of poisoning among patients aged 0-13 years at a paediatric hospital in Nairobi. *East African Medical Journal, 91*(11), 379-384.

Mutlu, M., Cansu, A., Karakas, T., Kalyoncu, M., & Erduran, E. (2010). Pattern of pediatric poisoning in the East Karadeniz region between 2002 and 2006: Increased suicide poisoning. *Hum Exp Toxicol, 29*(2), 131-6. doi:10.1177/0960327109357141

Narendra, B., Prava, B. J., & Kunal, P.Pharmacoepidemiology of common poisoning cases in children at a tertiary care teaching hospital, Odisha, India.

Nguefack, F., Chiabi, A., Ndounia, S. N., Bogne, J. B., Mah, E., Nguefack, S., & Fru, A. (2017). Clinical and epidemiologic study on unintentional domestic poisoning at the paediatric service of

the Yaounde Gynaeco-Obstetric and Pediatric Hospital. *The Journal of Medical Research, 3*(3), 164-168.

Obu, D. C., Orji, M. C., Muoneke, U. V., Asiegbu, U. V., & Ezegbe, G. O. (2020). Accidental childhood poisoning in paediatrics department of a tertiary care facility: A retrospective review. *Nigerian Journal of Paediatrics, 47*(3), 215-220.

Oguche, S., Bukbuk, D. N., & Watila, I. M. (2007). Pattern of hospital admissions of children with poisoning in the Sudano-Sahelian North Eastern Nigeria. *Nigerian Journal of Clinical Practice, 10*(2), 111-115.

Olatunya, O. S., Isinkaye, A. O., Ogundare, E. O., Oluwayemi, I. O., & Akinola, F. J. (2015). Childhood poisoning at a tertiary hospital in South West Nigeria. *Journal of Nepal Paediatric Society, 35*(2), 103-110. doi:http://dx.doi.org/10.3126/jnps.v35i2.12548

Oprescu, F., Peek-Asa, C., Wallis, A., Young, T., Nour, D., & Chereches, R. M. (2012). Pediatric poisonings and risk markers for hospital admission in a major emergency department in Romania. *Maternal and Child Health Journal, 16*(2), 495-500. doi:http://dx.doi.org/10.1007/s10995-011-0742-8

Oray, N. C., Hocaoglu, N., Oray, D., Demir, O., Atilla, R., & Tuncok, Y. (2008). Sedative-hypnotic medication exposures and poisonings in Izmir, Turkey. *Basic and Clinical Pharmacology and Toxicology, 103*(4), 380-385. doi:http://dx.doi.org/10.1111/j.1742-7843.2008.00303.x

Osaghae, D. O., & Sule, G. (2013). Accidental childhood poisoning in Benin city: Still a problem? *Journal of Medicine and Biomedical Research, 12*(2), 19-26. Retrieved from http://www.ajol.info/index.php/jmbr/article/view/104619/94672

Ozdemir, R., Bayrakci, B., Tekşam, O., Yalçin, B., & Kale, G. (2012). Thirty-three-year experience on childhood poisoning. *Turk J Pediatr, 54*(3), 251-9.

Ozdogan, H., Davutoglu, M., Bosnak, M., Tutanc, M., & Haspolat, K. (2008). Pediatric poisonings in southeast of Turkey: Epidemiological and clinical aspects. *Human and Experimental Toxicology, 27*(1), 45-48. doi:http://dx.doi.org/10.1177/0960327108088975

Paudyal, B. P. (2005). Poisoning: Pattern and profile of admitted cases in a hospital in central Nepal. *Journal of the Nepal Medical Association, 44*(159), 92-96.

Ram, P., Kanchan, T., & Unnikrishnan, B. (2014). Pattern of acute poisonings in children below 15 years - A study from Mangalore, South India. *Journal of Forensic and Legal Medicine, 25*, 26-29. doi:http://dx.doi.org/10.1016/j.jflm.2014.04.001

Raman, R., Kumar, S., & Muthukrishnan, L. (2015). A hospital-based epidemiologic study on acute pediatric poisonings in Chennai, India. *Asia Pacific Journal of Medical Toxicology, 4*(4), 156-160. Retrieved from http://apjmt.mums.ac.ir/article_6362_14f7792a050d808375851528f4628c44.pdf

Ramawat, P., & Jain, N. (2021). A study about clinic-epidemiological pattern of acute poisoning in pediatric age group. *Asian Journal of Medical Sciences, 12*(4), 48-53.

Rashid, M. M., Hasan, M. A., & Chowdhury, F. R. (2007). Childhood acute poisoning in a tertiary medical college hospital of Bangladesh. *Mymensingh Medical Journal : MMJ, 16*(2), 12.

Roy, M. P., Gupta, R., Bhatt, M., & Aggarwal, K. C. (2017). Profile of children hospitalized with acute poisoning in New Delhi. *Indian Pediatrics, 54*(3), 246-247.

Sadeghi-Bojd, S., & Khajeh, A. (2014). Chronological variations of children poisoning causes in Zahedan, south of Iran. *Int J High Risk Behav Addict, 3*(3), e19223. doi:10.5812/ijhrba.19223

Satish, K. V., Shivakumar, P., & Mugadlimath, A. (2016). A study of fatal and non-fatal paediatric poisoning cases in Bangalore, India. *Indian Journal of Forensic Medicine and Toxicology, 10*(1), 64-67. doi:http://dx.doi.org/10.5958/0973-9130.2016.00015.3

Sawalha, A. F. (2012). Deliberate self-poisoning: A study from Nablus. *Int J Adolesc Med Health, 24*(4), 373-7. doi:10.1515/ijamh.2012.054

Sharif, M. R., & Nouri, S. (2015). Clinical signs and symptoms and laboratory findings of methadone poisoning in children. *Iranian Journal of Pediatrics, 25*(1), e176. doi:http://dx.doi.org/10.5812/ijp.176

Sheikh, N. A., & Damodar, G. (2015). Spectrum of accidental paediatric poisoning at a tertiary care centre in South India. *Medico-Legal Update, 15*(1), 93-97. doi:http://dx.doi.org/10.5958/0974-1283.2015.00021.3

Sheriff, A., Rahim, A., Lailabi, M. P., & Gopi, J. (2011). Unintentional injuries among children admitted in a tertiary care hospital in north Kerala. *Indian Journal of Public Health, 55*(2), 125-127.

Shotar, A. M. (2005). Drug poisoning in childhood. *Saudi Medical Journal, 26*(12), 1948-1950. Retrieved from http://www.smj.org.sa/PDFFiles/Dec05/14Drug20050785.pdf

Shwe, D. D., Toma, B., Pate, S. I., Adedeji, I., & Oguche, S. (2013). Profile of hospital admissions of childhood poisoning at a North-Central Nigerian tertiary health care centre. *Jos Journal of Medicine, 7*(2)

Soori, H. (2001). Developmental risk factors for unintentional childhood poisoning. *Saudi Medical Journal, 22*(3), 227-230.

Suting, E., Bhaskar, V., & Batra, P. (2021). Changing epidemiology of poisoning in children: A retrospective study from a tertiary care center in New Delhi, India. *Indian Journal of Public Health, 65*(4), 400-402. doi:https://dx.doi.org/10.4103/ijph.IJPH_234_21

Tabrizi, M., Sarabi, S., Taramsari, M. R., Baghersalimi, A., Rad, A. H., & Darbandi, B. (2021). Changes in opioid poisoning pattern in children: A retrospective study in Rasht, Iran. *Iranian Journal of Toxicology, 15*(3), 151-156. doi:http://dx.doi.org/10.32598/IJT.15.3.794.1

Tarvadi, P. V., Bakkannavar, S. M., Manjunath, S., Palimar, V., Pradeep Kumar, G., & Shetty, M. (2013). Trends of poisoning among children at Kasturba Hospital, Manipal. *Nitte University Journal of Health Science, 3*(2), 25-28. Retrieved from http://nitte.edu.in/journal/june2013/TOPAC.pdf

Tirolla, R. M., Girotto, E., & Guidoni, C. M. (2021). Clinical and epidemiological analysis of suicide attempts in children assisted by a poison control center. *Rev.Paul.Pediatr.(Ed.Port., Online), 39*, e2019345. doi:10.1590/1984-0462/2021/39/2019345

Trangadia, M., Kharadi, R., & Gupta, B. D. (2016). Epidemiologic study of fatal and non-fatal poisoning case in pediatric, around Jamnagar region, Gujarat in India (january-december 2013). *International Journal of Medical Toxicology and Forensic Medicine, 6*(3), 128-134. Retrieved from http://journals.sbmu.ac.ir/ijmtfm/article/download/IJMTFM-10525/pdf-2

Ugwu, G. I. M., Okperi, B. O., Okolugbo, N. E., & Ugwu, E. N. (2012). Childhood poisoning in Warri, Niger Delta, Nigeria: A ten year retrospective study: Case studies. *African Journal of Primary Health Care and Family Medicine, 4*(1), 1-5.

Ulmeanu, C., & Nitescu Girnita, V. G. (2005). Mortality rate in acute poisoning in a pediatric toxicology department. *Przeglad Lekarski, 62*(6), 453-455.

Wasim, S., Agrawal, N., Pandita, N., Das, K., & Gupta, A. (2021). Clinical spectrum and severity of poisoning in the paediatric intensive care unit of a tertiary care center in Uttarakhand: A retrospective cohort study. *Journal of Clinical and Diagnostic Research, 15*(9), SC06-SC09. doi:http://dx.doi.org/10.7860/JCDR/2021/49611.15440

Yasmeen, M., Rehana, M., Hussain Bux, K., Kamran, Q., & Salma, S. (2010). *Clinical spectrum and outcome of accidental poisoning in children* Retrieved from https://pesquisa.bvsalud.org/gim/resource/en/emr-97908

Zarif, P., Uddin, S. Z., & Bashir, N. (2018). Analytical study of acute poisoning cases admitted in Lahore General Hospital, Lahore, Pakistan. *Medical Forum Monthly, 29*(6), 102-105. Retrieved from http://medforum.pk/images/pdf/2018/june2018.pdf
